# Glycosylation efficiencies on different solid supports using a hydrogenolysis-labile linker

**DOI:** 10.3762/bjoc.9.13

**Published:** 2013-01-16

**Authors:** Mayeul Collot, Steffen Eller, Markus Weishaupt, Peter H Seeberger

**Affiliations:** 1 Department of Biomolecular Systems, Max Planck Institute of Colloids and Interfaces, Am Mühlenberg 1, 14776 Potsdam, Germany; 2 Institut für Chemie und Biochemie, Freie Universität Berlin, Arnimallee 22, 14195 Berlin, Germany

**Keywords:** glycosylation, hydrogenolysis, linkers, oligosaccharides, resins, solid-phase synthesis

## Abstract

Automated oligosaccharide assembly requires suitable linkers to connect the first monosaccharide to a solid support. A new hydrogenolysis-labile linker that is stable under both acidic and basic conditions was designed, synthesized and coupled to different resins. Glycosylation and cleavage efficiencies on these functionalized solid supports were investigated, and restrictions for the choice of solid support for oligosaccharide synthesis were found.

## Findings

**Scheme 1:**
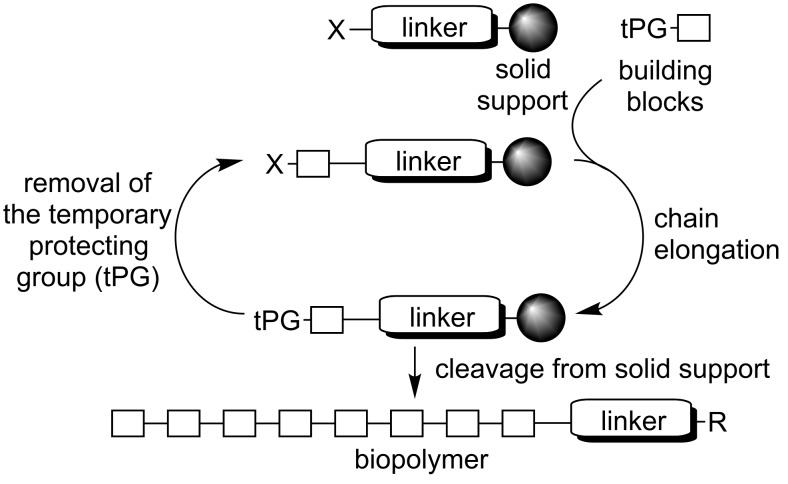
Solid-phase synthesis of biopolymers. X represents a reactive site such as an amino group for peptide synthesis or a hydroxy group for oligonucleotide and oligosaccharide synthesis.

Since Bruce Merrifield introduced the concept of solid-phase peptide synthesis in 1963 [1], synthesis on solid supports has evolved as a powerful tool for organic chemists [2]. Over the past fifty years this strategy has been successfully applied to the synthesis of other biopolymers, such as oligonucleotides [3] and oligosaccharides [4]. Solid-phase synthesis is performed on insoluble supports that are functionalized with a linker that connects the growing molecule with the resin (Scheme 1). Once the target molecule has been assembled, it is cleaved from the solid support. The solid-phase paradigm allows for the use of excess reagents to drive reactions to completion, as any leftovers are easily removed by washing of the resin between reaction steps.

Given the repetitive character of solid-supported synthesis, the process was successfully automated for all types of biopolymers [5–7]. As glycobiology is rapidly expanding [8], the need for synthetic tools has prompted synthetic carbohydrate chemists to develop methods for the accelerated synthesis of all types of glycans [9–19]. Automated synthesis of oligosaccharides is beginning to provide molecules for biological evaluation [20–23]. It was early on recognized that the linker plays a pivotal role for oligosaccharide synthesis, as its chemical properties determine the conditions that can be used for glycosylation and deprotection reactions [7,20,23–25]. Equally important is the choice of solid support and many different resins were briefly explored [26]. However, for automated solid-phase oligosaccharide synthesis, Merrifield polystyrene resin has almost exclusively been used as the solid support.

**Figure 1:**
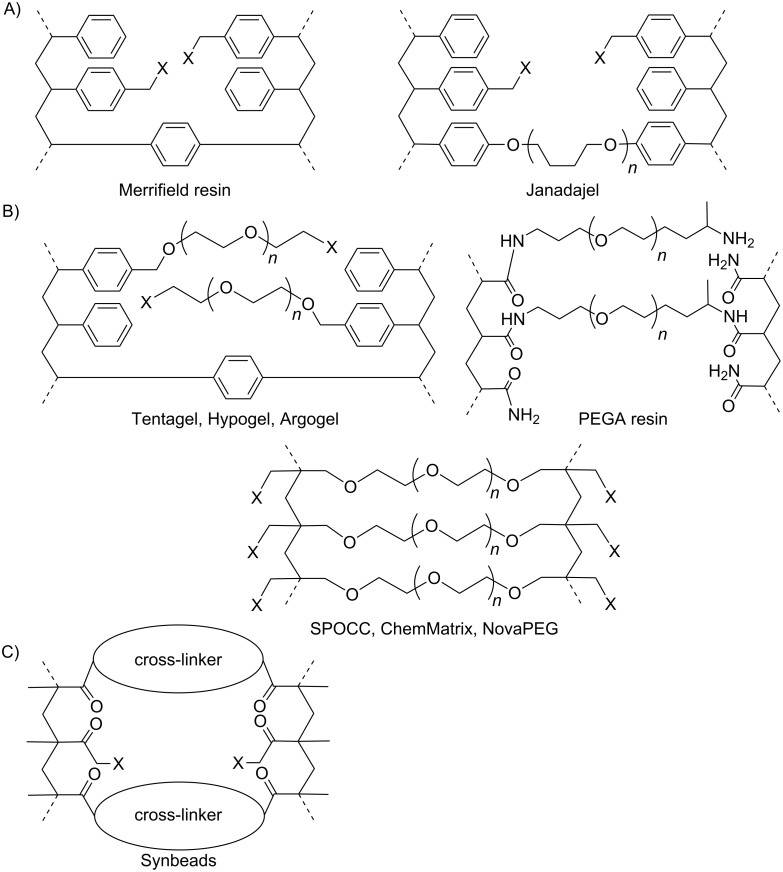
Different resins used for solid-phase synthesis. (A) Hydrophobic PS resins. (B) Water-compatible resins with PEG chains. (C) Nonswelling solid support compatible with aqueous reactions. X represents the functional groups, such as chlorides or amines.

Here, we describe the development of a new linker system that was tested in the context of different solid supports. In order to be suitable for automated solid-phase synthesis the resins have to be stable, chemically and mechanically, have to be permeable for the reagents, have to allow for reproducible loadings, and must exhibit good swelling behavior in a wide range of solvents. Mindful of these requirements, different solid supports have been developed (Figure 1, [27–28]). The most commonly used solid support for organic synthesis is the Merrifield resin [1]. This polystyrene (PS) resin shows good swelling properties in organic media but is not compatible with the aqueous conditions that are often required for hydrolysis or enzymatic reactions. There are different variations of pure polystyrene resins. Jandajel resin for example features crosslinking of PS chains by tetrahydrofuran derivatives [29–30]. To improve the swelling behavior of PS resins in polar solvents, the PS core was grafted with polyethylene glycol (PEG) chains [31], resulting in solid supports such as Tentagel, Hypogel or Argogel. These resins were successfully used for the synthesis of peptides [31]. Meldal and co-workers developed PEGA resins [32] with good swelling behavior in water and polar solvents. Since the amide bonds of this solid support mimic peptides, the degree of aggregation of peptide chains during solid-phase synthesis is decreased, which facilitates the synthesis of peptides and glycopeptides [33]. Since amides are incompatible with many organic reactions, pure PEG resins, such as SPOCC [34], ChemMatrix [35] or NovaPEG, were introduced. To perform enzymatic reactions on a solid support, nonswelling resins (Synbeads) with large surfaces and big cavities that can be accessed even by proteins were developed [36].

**Scheme 2:**
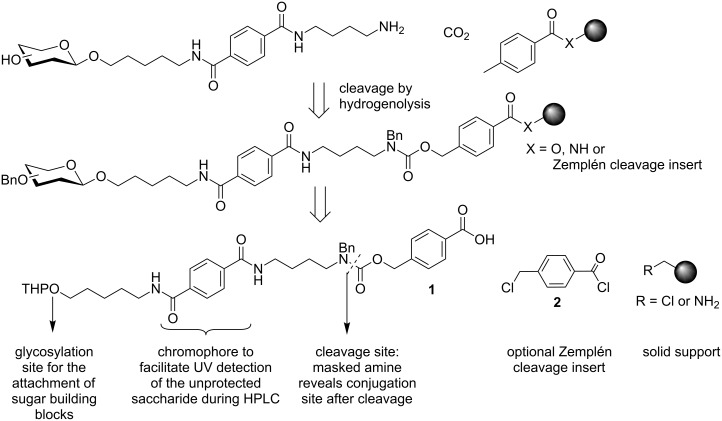
Design of linker **1**. Cleavage by hydrogenolysis from a solid support reveals a conjugation site for the synthesis of glycoconjugates or glycan arrays and simultaneously removes permanent benzyl protecting groups. The linker can be coupled to amino- and chloro-functionalized resins. By placement of insert **2** on amino resins, an additional Zemplén cleavage site for fast LC–MS analysis is introduced.

For the design of linkers for oligosaccharide synthesis on solid support, several key features have to be considered. Not only has the linker to be orthogonal to the reaction conditions necessary for chain elongation, which are in general acidic for glycosylations and basic for the removal of temporary protecting groups; the linker also has to allow for the introduction of many naturally occurring modifications such as sulfation or phosphorylation, as well as non-native features for biological experiments, such as linkers for glycoconjugation. This results in a high requirement concerning the chemical stability of the linker and solid support. Orthogonal linker **1** (Scheme 2) was designed to address these issues. Cleavage of the linker by hydrogenolysis results in a free amine functionality for the immobilization of oligosaccharides on glycan arrays or for the synthesis of glycoconjugates. Hydrogenolysis on a solid support has been used previously in peptide chemistry [37]. In the early 1980s, catalytic-transfer-hydrogenation conditions proved to be very efficient for both deprotection and cleavage of the peptide from the solid support [38]. In this context, in situ generation of palladium black by reduction of palladium(II) acetate with ammonium formate in DMF yielded the best results. Although hydrogenolysis is widely used in carbohydrate chemistry as a means to achieve the final deprotection step, few examples for hydrogenolytic cleavage of an oligosaccharide from a support have been reported [39–40]. To assemble oligosaccharides, linker **1** was equipped with a primary hydroxy group as a glycosylation site, which was obtained by removal of the tetrahydropyran (THP) protecting group after coupling to the solid support. A terephthalic chromophore was incorporated into the linker to facilitate the UV detection during HPLC analysis or purification. The linker was directly attached to chloro-functionalized resins leading to an ester linkage. Cleavage of this ester under Zemplén conditions provides quick access to samples for HPLC analysis that may be used to control the glycosylation efficiency during chain elongation. When amino-functionalized resins were used, insert **2** was placed to obtain the additional Zemplén cleavage site in addition to the stable amide linkage to the solid support. This construct was used for linker evaluation where rapid cleavage for HPLC analysis was of key importance. After the utility of the linker had been established the linker could be directly coupled to amino-functionalized resins, which resulted in an amide bond that is stable under Zemplén conditions.

**Scheme 3:**
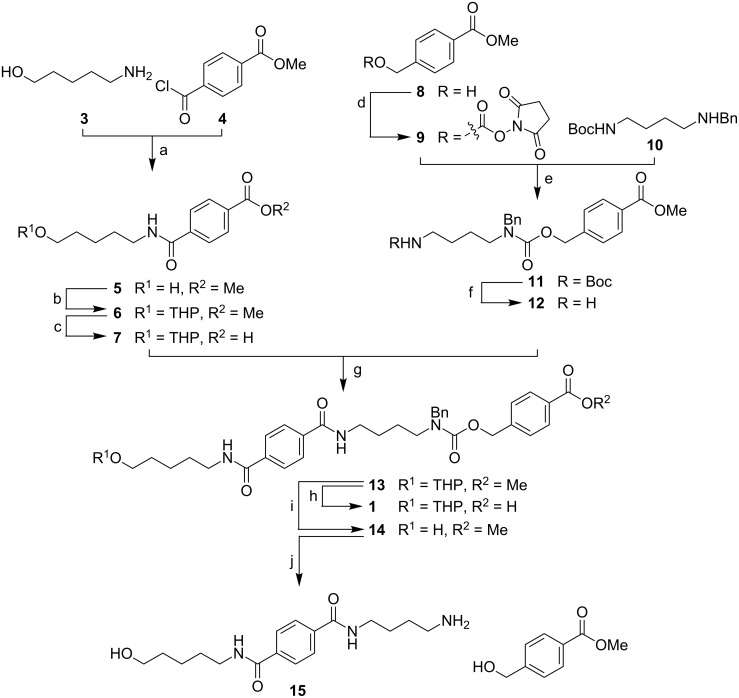
Synthesis of linker **1**. Reactions and conditions: (a) NEt_3_, DCM, rt, 84%; (b) DHP, pyridinium *p*-toluenesulfonate, DCM, rt, quant.; (c) 2 M aq NaOH, THF, rt, 91%; (d) DSC, NEt_3_, CH_3_CN, 0 °C to rt; (e) NEt_3_, DCM, rt, 80% over 2 steps; (f) TFA, DCM, rt, 99%; (g) NHS, DCC, DMAP, CH_3_CN, DCM, rt, 63%; (h) 2 M aq NaOH, THF, 55 °C, 92%; (i) *p*-TsOH·H_2_O, MeOH, DCM, rt, 94%; (j) Pd(OAc)_2_, HCOONH_4_, MeOH, H_2_O, 90%.

Linker **1** was prepared starting from chromophore fragment **7** and masked amine **12** (Scheme 3, Supporting Information File 1). Fragment **7** was synthesized starting from aminopentanol **3** and acyl chloride **4**. Following the condensation of **3** and **4**, the primary hydroxy group of the resulting intermediate **5** was protected and the ester was hydrolyzed to afford **7** in 76% yield over three steps. The synthesis of fragment **12** started with the transformation of **8** to carbonate **9**. Subsequent nucleophilic attack of secondary amine **10** [41–42] to afford intermediate **11** and removal of the Boc protecting group furnished amine **12**. Condensation of **7** and **12** provided precursor **13** in 63% yield. Finally, linker **1** was obtained by saponification of methyl ester **13**.

**Scheme 4:**
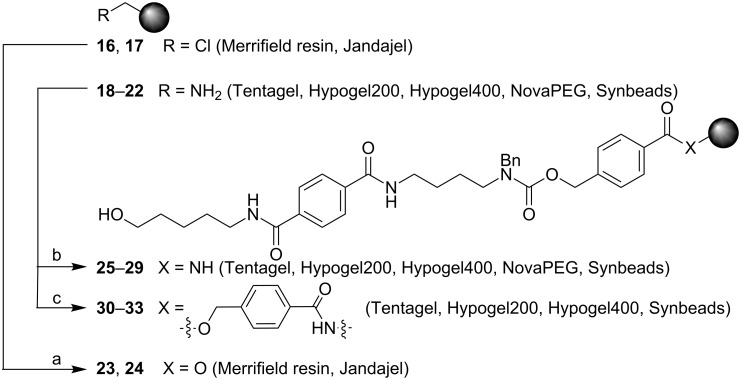
Coupling of linker **1** to different resins. Reactions and conditions: (a) 1. **1** and **16** or **17**, Cs_2_CO_3_, DMF, TBAI, 60 °C; 2. CsOAc, TBAI, DMF, 60 °C; 3. *p*-TsOH·H_2_O, MeOH, DCM, rt; (b) 1. **1** and **18**, **19**, **20**, **21** or **22**, HOBt, DIC, DMF, rt; 2. Ac_2_O, pyridine, DCM, rt; 3. *p*-TsOH·H_2_O, MeOH, DCM, rt; (c) 1. **2** and **18**, **19**, **20** or **22**, pyridine, DCM, rt; 2. Ac_2_O, pyridine, DCM, rt; 3. **1**, Cs_2_CO_3_, DMF, TBAI, 60 °C; 4. CsOAc, TBAI, DMF, 60 °C; 5. *p*-TsOH·H_2_O, MeOH, DCM, rt.

In the next step, solution-phase studies towards cleavage of linker **1** from a solid support were conducted. To this end, compound **14** was prepared and subjected to different conditions for hydrogenolysis (Scheme 3, Supporting Information File 1). Compound **14** was reduced by using palladium(II) acetate and ammonium formate. When the cleavage reaction was carried out in a mixture of methanol/ethyl acetate (3:2), *N*-methylation and *N*-formylation were observed (Supporting Information File 1). Considering prior evidence that methanol can generate formaldehyde in the presence of Pd(0) by an oxidative addition mechanism [43–44] and the observation that apolar solvents cause *N*-formylation during the hydrogenolysis reactions [45], our experimental results could be explained. To avoid any such side reactions, the hydrogenolytic cleavage was performed in MeOH and water resulting in pure **15** in 90% yield. Encouraged by the good cleavage result of model compound **14**, different solid supports were functionalized with linker **1** (Scheme 4). Coupling to both chloro-functionalized Merrifield resin **16** and Jandajel **17** was achieved by a tetrabutylammonium iodide (TBAI) mediated substitution in the presence of Cs_2_CO_3_. Capping of unreacted chlorides by cesium acetate and subsequent acidic hydrolysis of the THP protecting group led to ester-bound linkers **23** and **24**. Fluorenylmethoxycarbonyl (Fmoc) protection and deprotection of the hydroxy group of an aliquot enabled the determination of the loading by measurement of the UV absorption of the corresponding dibenzofulvene released upon Fmoc deprotection (Table 1, Supporting Information File 1, [46]).

**Table 1:** Functionalization of different resins with linker **1** and loading determination.

	chloro-functionalized PS resins		amino-functionalized water-compatible resins				

	Merrifield **16**	Jandajel **17**	Tentagel **18**	Hypogel200 **19**	Hypogel400 **20**	NovaPEG **21**	Synbeads **22**
initial loading [mmol/g]	0.74	1.00	0.30	0.92	0.71	0.66	0.70

	**23**	**24**	**25**	**26**	**27**	**28**	**29**
linker loading [mmol/g]	0.14	0.61	0.22	0.44	0.40	0.29	0.25
coupling efficiency	19%	61%	73%	48%	56%	44%	36%

			**30**	**31**	**32**		**33**
linker loading via insert [mmol/g]	–	–	0.13	0.23	0.21	–	0.05
coupling efficiency	–	–	43%	25%	30%	–	7%

Attachment of linker **1** to the amino-functionalized resins Tentagel (**18**), Hypogel200 (**19**), Hypogel400 (**20**), NovaPEG (**21**) and Synbeads (**22**) was achieved by dehydrative coupling in the presence of diisopropylcarbodiimide (DIC) and hydroxybenzotriazole (HOBt; Scheme 4). To avoid neutralization of the activator during glycosylation reactions, unreacted amino groups were capped by acetylation. Resin loadings with the amide-bound linkers **25**–**29** were determined by using variants including the ester insert for rapid cleavage (Table 1).

**Scheme 5:**
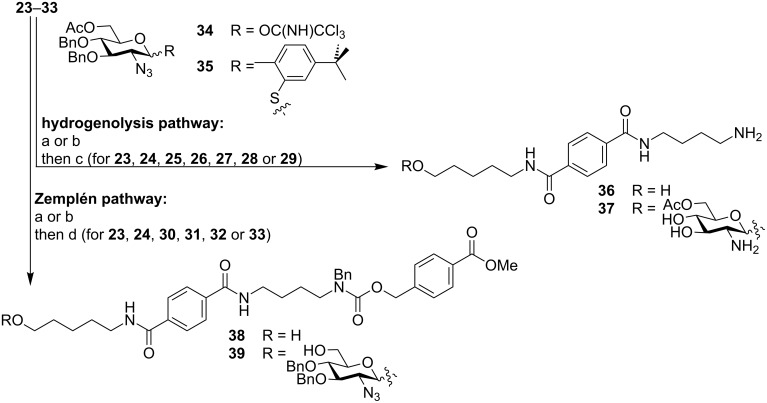
Model glycosylation by using an automated oligosaccharide synthesizer. Reactions and conditions: (a) **34**, TMSOTf, DCM, −15 °C (30 min); (b) **35**, NIS, TfOH, DCM, dioxane, different temperatures and reaction times; (c) Pd(OAc)_2_, HCOONH_4_, H_2_O; (d) NaOMe, MeOH, DCM, rt.

Glycosylation with monosaccharide building blocks **34** or **35** was performed by using an automated oligosaccharide synthesizer (Scheme 5, Supporting Information File 1). This synthesizer is an improved version of a recently disclosed synthesizer prototype [20] whereby a separate unit to accommodate aqueous chemistry was added. To avoid cross contamination of the anhydrous solutions that are used for glycosylation reactions, all aqueous solutions are completely separated from the organic units by an additional syringe pump. Building blocks **34** and **35** can be used for the synthesis of heparin, a major subclass of GAGs. The synthesis of heparin necessitates aqueous solutions to perform Staudinger reductions in the placement of amino groups as well as for ester saponification used to remove temporary protective groups prior to sulfation. A range of different glycosylation conditions were explored, whereby the couplings were performed either twice by using five equivalents of the building block each time or were carried out three times by using three equivalents of the building block each time. Glycosyl trichloroacetimidate **34** was activated by catalytic amounts of trimethylsilyl trifluoromethanesulfonate (TMSOTf) at −15 °C in dichloromethane or toluene. Thioglycoside **35** was activated with *N*-iodosuccinimide (NIS) and triflic acid (TfOH) in dichloromethane and dioxane. In order to establish optimal reaction conditions, temperatures ranging from −40 °C to 25 °C were screened and the reaction time was varied between 15 and 45 minutes.

After completion of the glycosylations, the products were cleaved from the resin by hydrogenolysis before the crude products were analyzed by LC–MS. In order to obtain high cleavage efficiencies and to ensure that all permanent protecting groups are removed during the cleavage process, an excess of Pd catalyst was used. Unfortunately, an efficient cleavage of the products from Merrifield resin was impossible since PS resins fail to swell in water. When dioxane was used to swell the PS resin, some partially deprotected compounds were detected. Other polar solvents that suppress the described side reactions and swell PS solid supports may have to be further investigated. Additionally, the suspension can be filtered and the solution can be resubmitted for a second hydrogenolysis reaction to remove the remaining protecting groups.

**Figure 2:**
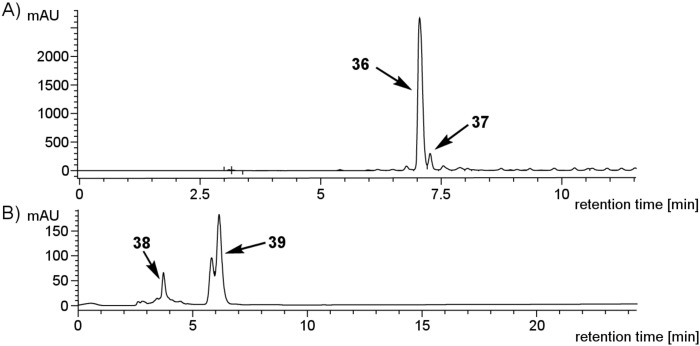
Representative HPLC chromatograms of glycosylation experiments on PS-based and water-compatible resins. (A) Tentagel (Nucleosil C4, 5% → 95% in 20 min, eluents: H_2_O and MeCN, detection at 254 nm). (B) Merrifield resin (Nucleosil C4, 50% → 80% in 30 min, eluents: H_2_O and MeCN, detection at 254 nm; the double peak for **39** is caused by an anomeric mixture).

On the other hand, when resins that are compatible with aqueous reaction conditions, such as Tentagel, were employed, glycosylation reactions proved to be ineffective and resulted in nonglycosylated linker **36** as the major product (Figure 2, A). A possible explanation for the low conversion to **37** is the long PEG chains contained in the resin structure that can either trap water to hydrolyze the monosaccharide building blocks or may complex the acidic activators due to the presence of many Lewis basic sites on PEG chains [47].

Since the hydrogenolytic linker cleavage did not work equally well for all types of solid support, this cleavage method was ill suited for the comparison of glycosylation efficiencies on different types of resin. Therefore, Zemplén conditions were employed as an alternative cleavage method. Sodium methoxide-mediated cleavage of the ester bond between linker **1** and the solid support in the case of polystyrene resins, as well as the ester bond between linker **1** and insert **2** in the case of all water-compatible resins, reliably afforded the crude products of the automated syntheses for analysis. For automated solid-phase syntheses on Merrifield resin, LC–MS analysis of the crude products indicated good glycosylation efficiencies. Only small quantities of nonglycosylated linker **38** were detected when compared to the desired product **39** (Figure 2B). It is well known that glycosylations on PS resins can be optimized to achieve full conversion [7,20–22]. However, due to the problems encountered regarding the hydrogenolytic linker cleavage on Merrifield resin, a further optimization of this system was not pursued.

To enable rapid LC–MS analysis and to exclude solubility issues caused by aqueous conditions during hydrogenolysis, the amino-functionalized resins **18**–**22** were equipped with an additional Zemplén cleavage site. To this end, insert **2** was coupled to these resins by amide-bond formation (Scheme 4, Supporting Information File 1). In the next step, the alkyl chlorides were displaced by the cesium carboxylate of **1**. Resin loadings were determined by Fmoc quantification (Table 1). Glycosylations on functionalized solid supports **30**–**33** were performed on the automated oligosaccharide synthesizer, and subsequent linker cleavage with sodium methoxide afforded the crude products, which were analyzed by LC–MS. These analyses clearly showed lower glycosylation efficiencies for all water-compatible resins when compared to PS resins **23** and **24**. To prevent the basic residues on the water-compatible resins from interfering with the acidic activators used during the glycosylations, solid supports **25**–**33** were washed before glycosylations with the acidic solutions. By using such prewashes, the ratio between the desired product **39** and the unglycosylated linker **38** improved, but complete conversions in glycosylations that are possible by using PS resins could not be achieved with water-compatible solid supports (data not shown).

**Scheme 6:**
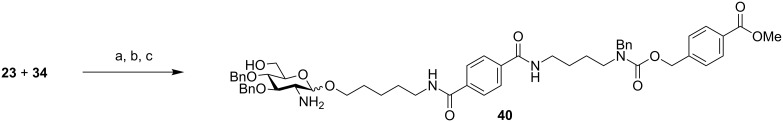
Glycosylation of **34** to linker **23** and subsequent Staudinger reduction of the azide. Reactions and conditions: (a) TMSOTf, DCM, −15 °C, 30 min; (b) PMe_3_, NEt_3_, H_2_O, THF, 25 °C, 30 min; (c) NaOMe, MeOH, DCM, rt.

To investigate the orthogonality of the linker for the introduction of naturally occurring modifications in oligosaccharides, the azide protecting group of the glucosamine was reduced under Staudinger conditions (Scheme 6, Supporting Information File 1). Therefore, imidate **34** was glycosylated to functionalized resin **23**. In the next step the azide was reduced by using PMe_3_ under basic and aqueous conditions. The use of THF swelled the PS resin, granting access to the reactive sites on the solid support. The azide reduction can be used as a key step to facilitate *N*-sulfation, which is necessary in the synthesis of heparin [48], or for the introduction of prevalent *N*-acetates.

In summary, we demonstrate that PS-based resins perform best in the automated solid-phase oligosaccharide synthesis. A linker that can be cleaved by hydrogenolysis and incorporates a chromophore to facilitate LC–MS analysis and purification was developed and served as example for glycosylation studies involving different solid supports. Cleavage of this linker is more efficient in aqueous media and necessitates the use of PEG-containing resins for the best results.

## Supporting Information

File 1 Experimental details, characterization data and spectra.
