# Supplementary material for: Glycosylation efficiencies on different solid supports using a hydrogenolysis-labile linker
Source: Beilstein J Org Chem. 2013 Jan 16;9:97–105. doi: 10.3762/bjoc.9.13 (PMC3566856; doi:10.3762/bjoc.9.13)
Supplement: File 1 — Experimental details, characterization data and spectra. [file Beilstein_J_Org_Chem-09-97-s001.pdf]

# Supporting Information

## for

# Glycosylation efficiencies on different solid supports using a hydrogenolysis-labile linker

Mayeul Collot[1,2], Steffen Eller[1,2], Markus Weishaupt[1,2] and Peter H. Seeberger[*1,2]

Address: [1]Department of Biomolecular Systems, Max Planck Institute of Colloids and Interfaces, Am Mühlenberg 1, 14776 Potsdam, Germany and [2]Institut für Chemie und Biochemie, Freie Universität Berlin, Arnimallee 22, 14195 Berlin, Germany.

Email: Peter H. Seeberger - peter.seeberger@mpikg.mpg.de

*Corresponding author

### Experimental details, characterization data and spectra

1. General information for chemical synthesis.......S2
2. Synthesis of linker **1**.......S3
3. Preliminary cleavage investigations.......S8
4. Resin functionalization.......S10
5. Loading determination.......S11
6. Glycosylation modules for glycosylations using the automated synthesizer.......S12
7. Detailed glycosylation procedures (synthesis of compounds **36–40**).......S13
8. Spectra of new compounds.......S16

## 1. General information for chemical synthesis

All chemicals used were reagent grade and used as supplied, except where noted. Molecular sieves were activated prior to use by being heated under high vacuum. All reactions were performed in oven-dried glassware under an argon atmosphere, unless noted otherwise. *N*,*N*-Dimethylformamide (DMF), dichloromethane (DCM), toluene and tetrahydrofuran (THF) were purified by a Cycle-Tainer Solvent Delivery System, unless noted otherwise. Analytical thin-layer chromatography (tlc) was performed on Merck silica gel 60 F254 plates (0.25 mm). Compounds were visualized by UV irradiation or by dipping the plate in a cerium sulfate ammonium molybdate (CAM) solution or in a 1:1 mixture of $H_2SO_4$ (2 N) and resorcine monomethylether (0.2%) in ethanol. Flash column chromatography was carried out using forced flow of the indicated solvent on Fluka Kieselgel 60 (230–400 mesh). All automated glycosylations were performed on an automated oligosaccharide synthesizer prototype with either anhydrous solvents of the Cycle-Tainer Solvent Delivery System or commercially available anhydrous solvents (Acros Organics: AcroSeal Dry Solvents). LC–MS chromatograms were recorded on an Agilent 1100 Series spectrometer. Loading determination of functionalized resins was obtained using a Shimadzu UV-MINI-1240 UV spectrometer. $^{1}$H NMR, $^{13}$C NMR spectra were recorded on a Varian Mercury 400 (400 MHz) or 600 (600 MHz) spectrometer in $CDCl_3$ or $CD_3OD$ with chemical shifts referenced to internal standards ($CDCl_3$: 7.26 ppm $^{1}$H, 77.0 ppm $^{13}$C) unless stated otherwise. Splitting patterns are indicated as s, singlet; d, doublet; t, triplet; q, quartet; m, multiplet; brs, broad singlet for $^{1}$H NMR data. NMR chemical shifts (δ) are reported in ppm and coupling constants (*J*) are reported in Hz. High-resolution mass spectral (HRMS) analyses were performed by the MS-service in the Department of Organic Chemistry at Free University Berlin using an Agilent 6210 ESI-TOF (Agilent Technologies, Santa Clara, CA, USA).

## 2. Synthesis of linker 1

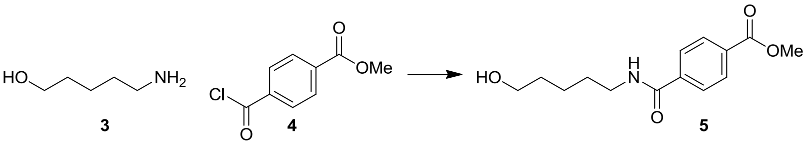


To a solution of 4-(methoxycarbonyl)benzoic acid (10.0 g, 55.55 mmol) in anhydrous THF (10 mL), oxalyl chloride (9.5 mL, 111.1 mmol, 2 equiv) was added under bubbler control. When the bubbling was slow and regular, a drop of DMF was carefully added to the solution. After 1 h the mixture was evaporated, and the crude acyl chloride **4** was dissolved in anhydrous DCM (30 mL) under an argon atmosphere. This solution was slowly added to a mixture of 5-amino-1-pentanol (**3**) (8.3 mL, 83.32 mmol, 1.5 equiv) and triethylamine (23 mL, 166.6 mmol, 3 equiv) in dry DCM (60 mL) under argon. After 30 min the solvents were evaporated to give a yellow solid, which was dissolved in DCM and washed with 1 M HCl and saturated $NaHCO_3$ solution, and then the organic layer was dried over $MgSO_4$ and filtered, and the solvent was evaporated. The crude product was recrystallized from EtOAc. The white crystals were collected and dried under vacuum to give **5** (12.3 g, 84%). $R_f$ 0.41 (DCM/MeOH: 9/1); mp: 117 °C; $^1$H NMR (500 MHz, $CDCl_3$): δ 8.05 (d, $J$ = 8.5 Hz, 2H, Ha), 7.80 (d, $J$ = 8.4 Hz, 2H, Hb), 6.52 (s, 1H, NH), 3.92 (s, 3H, OMe), 3.64 (t, $J$ = 6.3 Hz, 2H, $H_5$), 3.45 (q, $J$ = 6.6 Hz, 2H, $H_1$), 1.91 (s, 1H, OH), 1.62 (m, 4H, $H_2$, $H_4$), 1.45 (m, 2H, $H_3$);$^{13}$C NMR (125 MHz, $CDCl_3$): δ 166.8 (CO), 166.4 (CO), 138.7 (Cq Ar), 132.6 (Cq Ar), 129.8 (Cb), 127.0 (Ca), 62 .5 (OMe), 52.4 ($C_5$), 40.1 ($C_1$), 32.1 ($C_2$ or $C_4$), 29.3 ($C_2$ or $C_4$), 23.2 ($C_3$); HRMS–ESI ($m/z$): $[M + Na]^+$ calcd for $C_{14}H_{19}NNaO_4$, 288.1212; found, 288.1214.

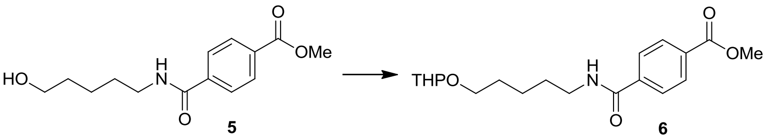


To a mixture of **5** (4.77 g, 18.0 mmol) and 3,4-dihydro-2*H*-pyran (3.27 mL, 36.0 mmol, 2 equiv) in anhydrous DCM pyridinium *p*-toluenesulfonate (0.90 g, 3.60 mmol, 0.2 equiv) was added and the solution was stirred overnight. The reaction was quenched by the addition of a saturated $NaHCO_3$ solution, the phases were separated, the organic layer was dried over $MgSO_4$ and filtered, and the solvent was removed in vacuo to give **6** (6.28 g, quant.) as colorless oil. $R_f$ 0.57 (DCM/MeOH: 95/5); $^1$H NMR (400 MHz, $CDCl_3$): δ 8.04 (d, $J$ = 8.2 Hz, 2H, Ha), 7.79 (d, $J$ = 8.2 Hz, 2H, Hb), 6.43 (s, 1H, NH), 4.53-4.52 (m, 1H, $H_6$), 3.91 (s, 3H,

OMe), 3.82 (td, $J$ = 9.7, 3.6 Hz, 1H, OCH), 3.73 (dt, $J$ = 9.6, 6.6 Hz, 1H, OCH), 3.42 (m, 4H, 2 OCH, $H_1$), 1.77 (m, 1H, $CH_2$), 1.63 (m, 5H, $CH_2$), 1.47 (m, 6H, $CH_2$); $^{13}C$ NMR (100 MHz, $CDCl_3$): δ 164.0 (CO), 163.7 (CO), 136.1 (Cq Ar), 129.9 (Cq Ar), 127.1 (Cb), 124.2 (Ca), 96.4 ($C_6$), 64.7 ($C_5$ or $C_{10}$), 59.9 ($C_5$ or $C_{10}$), 49.7 (OMe), 37.5 ($C_1$), 28.1 ($CH_2$), 26.7 ($CH_2$), 26.6 ($CH_2$), 22.8 ($CH_2$), 21.1 ($CH_2$), 17.1 ($CH_2$); HRMS–ESI (*m*/*z*): $[M + Na]^+$ calcd for $C_{19}H_{27}NNaO_5$, 372.1787; found, 372.1795.

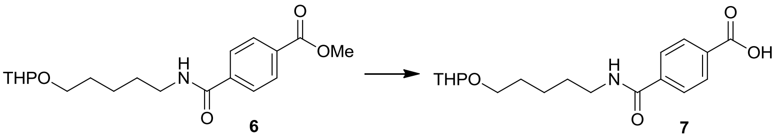


To a solution of **6** (6.28 g, 18.0 mmol) in THF (150 mL), a 2 M aqueous NaOH solution (45.0 mL, 90.0 mmol, 5 equiv) was added. The heterogeneous solution was stirred overnight. The pH was adjusted to 6–7 with an aqueous 1 M HCl solution. The organic solvent was evaporated and the precipitate was filtered off and collected, washed with water and dried under vacuum to give **7** (5.50 g, 91%) as a white solid. $R_f$ 0.31 (DCM/MeOH: 95/5); $^1H$ NMR (400 MHz, $CD_3OD$): δ 8.08 (d, $J$ = 8.4 Hz, 2H, Ha), 7.88 (d, $J$ = 8.4 Hz, 2H, Hb), 4.56 (t, $J$ = 3.7 Hz, 1H, $H_6$), 3.83 (ddd, $J$ = 11.3, 8.1, 3.2 Hz, 1H, OCH), 3.73 (dt, $J$ = 9.6, 6.5 Hz, 1H, OCH), 3.50-3.37 (m, 4H, OCH, $H_1$), 1.78 (dd, $J$ = 8.6, 6.4 Hz, 1H, $CH_2$), 1.69-1.60 (m, 5H, $CH_2$), 1.56-1.43 (m, 6H, $CH_2$); $^{13}C$ NMR (100 MHz, $CD_3OD$): δ 167.8 (COOH), 167.4 (CO amide), 138.4 (Cq Ar), 133.1 (Cq Ar), 129.4 (C Ar), 126.9 (C Ar), 98.8 (C6), 67.0 ($CH_2O$), 61.9 ($CH_2O$), 39.6 ($C_1$), 30.4 ($CH_2$), 29.0 ($CH_2$), 28.7 ($CH_2$), 25.1 ($CH_2$), 23.3 ($CH_2$), 19.2 ($CH_2$). HRMS–ESI (*m*/*z*): $[M + Na]^+$ calcd for $C_{18}H_{25}NNaO_5$, 358.1630; found, 358.1623.

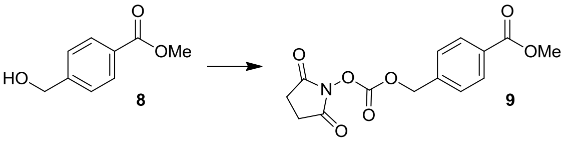


To a mixture of methyl 4-(hydroxymethyl)benzoate (**8**) (1.16 g, 6.99 mmol) and triethylamine (2.91 mL, 20.96 mmol, 3 equiv) in anhydrous acetonitrile (40 mL), *N*,*N*'-disuccinimidyl carbonate (2.68 g, 10.48 mmol, 1.5 equiv) was added at 0 °C. After 5 min the heterogeneous solution was allowed to warm to room temperature. After 30 min the homogeneous mixture was extracted with DCM and washed with a saturated $NaHCO_3$ solution, the organic layer was dried over $MgSO_4$ and filtered, and the solvent was evaporated to give **9** as a yellow syrup, which was used for the next step without any further purification.

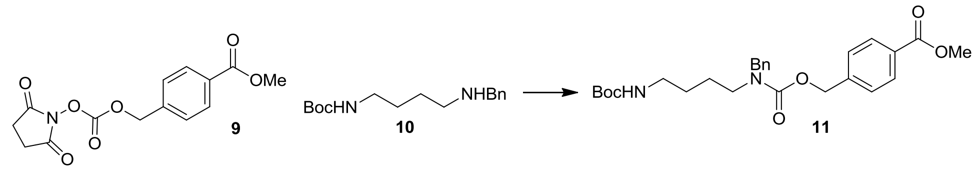

Compound **9** was dissolved in 20 mL of anhyrous DCM and slowly added to a mixture of **10** (1.94 g, 6.99 mmol, 1 equiv) and triethylamine (1.45 mL, 10.48 mmol, 1.5 equiv) in anhydrous DCM (50 mL). After 5 min, when tlc analysis indicated complete conversion of the starting material, the solution was washed with a saturated $NaHCO_3$ solution, the organic layer was dried over $MgSO_4$ and filtered, and the solvent was evaporated. The residue was purified by column chromatography on silica gel (cyclohexane/EtOAc: 7/3) to give **11** (2.63 g, 80%) as white solid. $R_f$ 0.42 (cyclohexane/ EtOAc: 7/3); $^1$H NMR (600 MHz, $CDCl_3$) Mixture of rotamers: δ 8.00 (2d, 2H, H Ar), 7.44-7.18 (m, 7H, 2H Ar, 5H Bn), 5.23 (2s, 2H, $CH_2$ Cbz), 4.64 (broad s, 1H, NH), 4.51 (s, 2H, $CH_2$ Bn), 3.92 (2s, 3H, OMe), 3.31 (broad s, 1H, $NCH_2$), 3.24 (broad s, 1H, $NCH_2$), 3.11-3.05 (2 broad s, 2H, $NCH_2$), 1.57-1.44 (m, 13H, tBu, $2CH_2$); $^{13}$C NMR (175 MHz, $CDCl_3$): δ 166.7 (CO ester), 156.4 (CO), 155.9 (CO), 141.9 (Cq Ar), 137.7 (Cq Ar), 129.8-127.0 (C Ar), 66.5 (CH2 Cbz), 52.1 (OMe), 50.6 (CH2 Bn), 47.1 ($NCH_2$), 45.9 ($NCH_2$), 40.1 ($NCH_2$), 29.7 (Cq tBu), 28.4 ($CH_3$ tBu), 27.3 ($CH_2$), 26.9 ($CH_2$), 25.4 ($CH_2$), 25.0 ($CH_2$); HRMS–ESI (*m*/*z*): $[M + Na]^+$ calcd for $C_{26}H_{34}N_2NaO_6$, 493.2315; found, 493.2317.

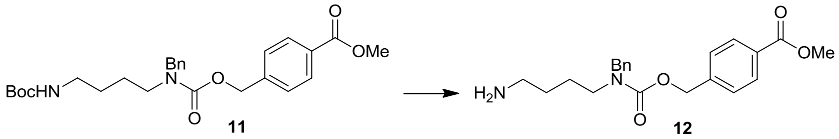

To a solution of **11** (3.59 g, 7.64 mmol) in DCM (40 mL) was added trifluoroacetic acid (5.67 mL, 76.38 mmol, 10 equiv). The reaction was stirred at room temperature until the starting material was no longer detectable by tlc. The pH of the solution was adjusted to nearly 14 by addition of an aqueous solution of 1 M NaOH. The product was extracted with DCM. The organic layer was dried over $MgSO_4$ and filtered, and the solvent was removed in vacuo to give **12** (2.80 g, 99%) as a yellow syrup, which was directly used in the next step.

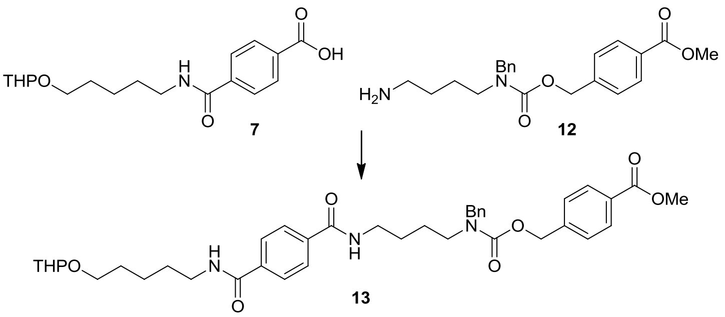


To a mixture of **7** (4.29 g, 12.79 mmol) and *N*-hydroxysuccinimide (2.21 g, 19.18 mmol, 1.5 equiv) in acetonitrile (30 mL) DCC (3.95 g, 19.18 mmol, 1.5 equiv) was added. After 15 min the amine **12** (4.73 g, 12.79 mmol, 1 equiv) was dissolved in 30 mL of DCM and added to the mixture. After 15 min DMAP (2.34 g, 19.18 mmol, 1.5 equiv) was added and the reaction was stirred overnight. The solution was filtered through celite and washed successively with 1 M HCl and a saturated $NaHCO_3$ solution, the organic layer was dried over $MgSO_4$ and filtered, and the solvents were evaporated. The residue was purified by column chromatography on silica gel (cyclohexane/EtOAc: 8/2) to give **13** (5.59 g, 63%) as colorless syrup. $R_f$ 0.29 (cyclohexane/EtOAc: 2/8); $^1$H NMR (400 MHz, $CDCl_3$) mixture of rotamers: δ 7.88 (d, *J* = 8.2 Hz, 2H, H Ar), 7.66 (m, 4H, H Ar), 7.31-7.09 (m, 7H, H Ar), 6.94 (broad s, NH), 6.71-6.57 (3 broad s, NH), 5.12 (s, 2H, $CH_2$ Cbz), 4.47-4.42 (m, 3H, OCH THP, $CH_2$ Bn), 3.82-3.74 (m, 4H, OMe, OCH THP), 3.68-3.64 (m, 1H, OCH), 3.42-3.19 (m, 8H, 3$NCH_2$, OCH, OCH THP), 1.74-1.37 (m, 16H); $^{13}$C NMR (100 MHz, $CDCl_3$) mixture of rotamers : δ 166.8 (CO), 166.8 (CO), 156.4 (CO), 156.1 (CO), 141.9 (C Ar), 141.6 (C Ar), 137.5 (C Ar), 137.2 (C Ar), 137.0 (C Ar), 129.7 (C Ar), 129.6 (C Ar), 128.6 (C Ar), 127.4 (C Ar), 127.2 (C Ar), 127.2 (C Ar), 127.0 (C Ar), 99.0 (OCH THP), 67.3 ($OCH_2$ THP), 66.4 ($CH_2$ Cbz), 62.55 ($OCH_2$), 52.1 (OMe), 50.6 ($CH_2$ Bn), 46.0 ($NCH_2$), 40.1 ($NCH_2$), 39.6, 30.7, 29.3, 29.2, 25.4, 23.7, 19.7; HRMS–ESI (*m*/*z*): $[M + Na]^+$ calcd for $C_{39}H_{49}N_3NaO_8$, 710.3417; found, 710.3412.

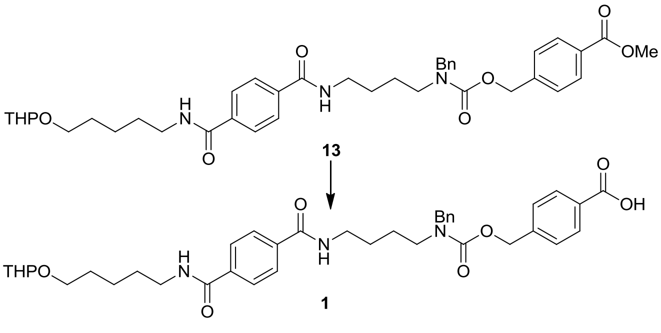

To a solution of **13** (0.15 g, 0.22 mmol) in THF (2 mL), a 2 M aqueous solution of NaOH (0.55 mL, 1.090 mmol, 5 equiv) was added, and the mixture was stirred overnight at 55 °C. The solution was cooled down to room temperature and the pH was adjusted to 7–8 by addition of IR 120 $H^+$ resin. The resin was filtered off and washed with MeOH. The solvents were evaporated and the crude compound was co-evaporated with toluene. The residue was purified by column chromatography on silica gel (DCM/MeOH: 95/5) to give **1** (0.14 g, 92%) as a white foam. $R_f$ 0.16 (DCM/MeOH: 95/5); $^1$H NMR (400 MHz, $CDCl_3$) mixture of rotamers: δ 7.88 (m, 2H, 2H Ar), 7.64 (m, 4H, 4 H Ar), 7.29-7.08 (m, 7H, H Ar), 6.87 (2 broad s, 2H, 2NH), 5.12 (s, 2H, $CH_2$ Cbz), 4.48 (m, 1H, OCH THP), 4.41 (2s, 2H, $CH_2$ Bn), 3.77 (td, $J$ = 7.4, 3.6 Hz, 1H, OCH), 3.65 (dt, $J$ = 9.6, 6.7 Hz, 1H, OCH THP), 3.43-3.17 (m, 8H, 3N$CH_2$, OCH THP, OCH), 1.70-1.37 (m, 16H, $CH_2$); $^{13}$C NMR (100 MHz, $CDCl_3$) mixture of rotamers: δ 169.4 (CO), 167.1 (CO), 167.1 (CO), 167.0 (CO), 156.2 (CO Cbz), 142.1 (C Ar), 137.4 (C Ar), 130.1 (C Ar), 128.6 (C Ar), 127.4 (C Ar), 127.3 (C Ar), 127.2 (C Ar), 127.1 (C Ar), 127.0 (C Ar), 99.0 (OCH THP), 67.4 (O$CH_2$ THP), 66.5 ($CH_2$ Cbz), 62.5 (O$CH_2$), 50.6 ($CH_2$ Bn), 46.7, 46.0, 40.1, 39.7, 30.7, 29.6, 29.2, 29.1, 29.1, 29.1, 29.0, 26.8, 26.8, 26.1, 26.0, 25.6, 25.3, 25.3, 25.3, 25.2, 25.1, 23.6, 19.7; HRMS–ESI ($m/z$): $[M + Na]^+$ calcd for $C_{38}H_{47}N_3NaO_8$, 696.3261; found, 696.3269.

## 3. Preliminary cleavage investigations

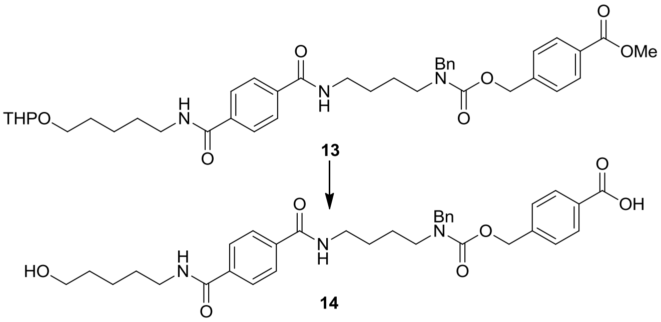

To a solution of **13** (0.280 g, 0.407 mmol) in methanol (5 mL) *p*-TsOH·$H_2O$ (0.007 g, 0.040 mmol, 0.1 equiv) was added. After 1 h basic resin IRA 67 was added to neutralize the solution. The solution was filtered off, the filtrate was evaporated, and the crude was purified by column chromatography on silica gel (EtOAc) to give 0.230 g (94%) of **14** as colorless syrup. $R_f$ 0.40 (DCM/MeOH : 95/5); $^1$H NMR (400 MHz, $CDCl_3$) mixture of rotamers: δ 7.86-7.84 (m, 2H, H Ar), 7.63 (s, 4H, H Ar), 7.36-7.06 (m, 7H, H Ar), 5.08 (s, 2H, $CH_2$ Cbz), 4.40 (s, 2H, $CH_2$ Bn), 3.80 (s, 3H, OMe), 3.49 (t, *J* = 6.3 Hz, 2H, $CH_2$OH), 3.26 (td, *J* = 18.4, 6.3 Hz, 6H, 3 $CH_2$-N), 2.89 (s, 1H, OH), 1.47 (td, *J* = 15.0, 7.8 Hz, 8H, $CH_2$), 1.31 (d, *J* = 6.8 Hz, 2H, $CH_2$); $^{13}$C NMR (100 MHz, $CDCl_3$): δ 167.2 (CO), 167.1 (CO), 166.8 (CO), 156.4 (CO), 156.4 (CO), 156.1 (CO), 141.9 (C Ar), 141.6 (C Ar), 137.4 (C Ar), 137.0 (C Ar), 129.7 (C Ar), 129.5 (C Ar), 128.6 (C Ar), 127.4 (C Ar), 127.2 (C Ar), 127.1 (C Ar), 127.0 (C Ar), 66.4($CH_2$ Cbz), 62.2 ($CH_2$OH), 60.3, 52.1 (OMe), 50.4 ($CH_2$ Bn), 46.9 ($CH_2$N), 46.0 ($CH_2$N), 46.0 ($CH_2$N), 40.0 ($CH_2$N), 39.6 ($CH_2$N), 32.0 ($CH_2$), 29.1 ($CH_2$), 26.8 ($CH_2$), 26.8 ($CH_2$), 26.3 ($CH_2$), 25.6 ($CH_2$), 25.6 ($CH_2$), 25.6 ($CH_2$), 25.2 ($CH_2$), 23.1 ($CH_2$), 21.0 ($CH_2$), 14.1 ($CH_2$); HRMS–ESI (*m*/*z*): $[M + Na]^+$ calcd for $C_{34}H_{41}N_3NaO_7$, 626.2842; found, 626.2834.

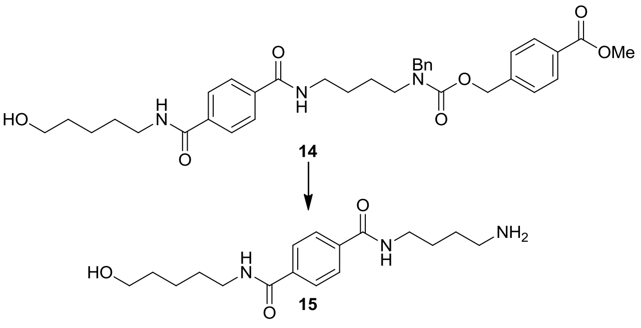

To a solution of **14** (0.060 g, 0.1 mmol) in a mixture of water (3 mL) methanol (1 mL) palladium acetate (0.022 g, 0.1 mmol, 1 equiv) and ammonium formate (0.012 g, 0.2 mmol, 2 equiv) were added. The reaction mixture quickly turned black and was stirred at room temperature overnight. The solution was filtered through celite, which was washed with methanol and water. The filtrate was evaporated and the white precipitate was filtered off. The filtrate was evaporated to give 29 mg of **12** (90%) as a white solid. HPLC showed purity over 95%. $^{1}$H NMR (400 MHz, $D_2O$): δ 8.32 (s, 1H), 7.67 (s, 4H, H Ar), 3.47 (t, *J* = 6.5 Hz, 2H, $CH_2OH$), 3.32-3.24 (m, 4H, $CH_2NHCO$), 2.92 (t, *J* = 6.3 Hz, 2H, $CH_2NH_2$), 1.59-1.44 (m, 8H, $CH_2$), 1.29-1.26 (m, 2H, $CH_2$); ESIMS *m/z*: $[M + H]^+$ calcd for $C_{17}H_{28}N_3O_3$, 322.2; found, 322.3.

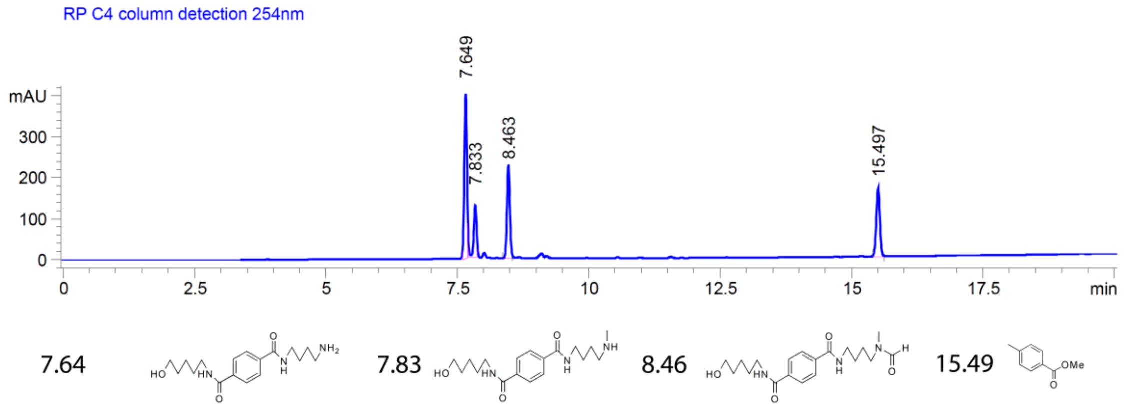


**Figure S1:** Chromatogram: CTH cleavage of **14** in methanol/ethyl acetate (3/2).

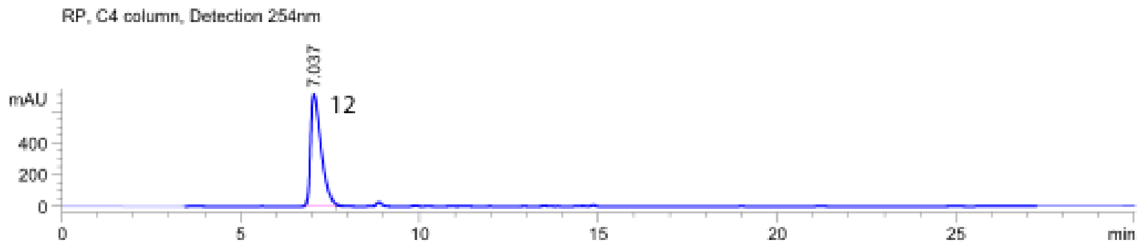


**Figure S2:** Chromatogram: CTH cleavage of **14** in methanol/water (1/3).

## 4. Resin functionalization

4.1. Synthesis of resins **23** and **24**

The resins **16** and **17** were swollen in DCM in a flask overnight and gently shaken on a rotavap. The solvent was drained and a solution of **1** (1.2 equiv) in DMF (ca. 0.2 M), $Cs_2CO_3$ (1.5 equiv) and TBAI (1 equiv) were added. The solution was shaken at 60 °C and 200 mbar on the rotary evaporator overnight. The resins were washed successively with DMF/water (1/1), DMF, MeOH, DCM, MeOH, DCM and swollen in DCM for 1 h. The resins were placed in a flask with DMF and CsOAc (2 equiv) and TBAI (1 equiv) were added. The solution was stirred at 60 °C and 200 mbar on the rotavap overnight. The resins were washed successively with DMF/water (1/1), DMF, MeOH, DCM, MeOH, DCM. The resins were shaken overnight in a 0.03 M solution of *p*-TsOH·$H_2O$ in DCM/methanol (1/1). The solution was drained, and the resins were washed with DCM, MeOH and DCM and dried under vacuum.

4.2. Synthesis of resins **25**–**29**

The amino-functionalized resins (**18**–**22**) were swollen in DCM overnight. The solvent was drained, and a solution of **1** (1.2 equiv) in DMF (ca. 0.2 M), and HOBt (6 equiv) and DIC (6 equiv) were added. The resins were shaken overnight at room temperature and washed with DCM, MeOH and DCM. DCM/pyridine/$Ac_2O$ (2/1/1) were added, and the resins were shaken overnight and washed with DCM, MeOH, DCM and a 0.03 M solution of *p*-TsOH·$H_2O$ in DCM/methanol (1/1) to neutralize the remaining pyridine. The resins were shaken overnight in a 0.03 M solution of *p*-TsOH·$H_2O$ in DCM/methanol (1/1). The solution was drained and the resins were washed with DCM, MeOH and DCM before being dried under vacuum.

4.3. Synthesis of resin **30**–**33**

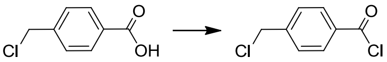


**2**

Oxalyl chloride (5 mL) was added to (chloromethyl)benzoic acid (2.0 g, 11.76 mmol) under bubbler control. A drop of DMF was carefully added and the reaction was stirred at room temperature overnight. The solvents were evaporated to give **2** (2.2 g, 99%) as a syrup, which was used in the next step without any further purification. $^1$H NMR (400 MHz, $CDCl_3$): δ 8.12 (d, *J* = 8.4 Hz, 2H, H Ar), 7.54 (d, *J* = 8.5 Hz, 2H, H Ar), 4.63 (s, 2H, $CH_2$).

The amino-functionalized resins (**18**, **19**, **20** and **22**) were swollen in DCM overnight. The solvent was drained and a solution of **2** (2 equiv) in DCM/pyridine (3:2; ca. 0.2 M) was added. The resins were shaken overnight at room temperature, and washed with DCM, MeOH and DCM. DCM/pyridine/$Ac_2O$ (2/1/1) were added, and the resins were shaken overnight and washed with DCM, MeOH, DCM. The resins were functionalized with **1** following the same procedure as described above.

## 5. Loading determination

Dried resin (ca. 50 mg) was placed in a syringe (5 mL) equipped with a frit. DCM (2–3 mL) was added to swell the resin for 1 h. The DCM was drained, and a solution of FmocCl (approx. 20 equiv according to the initial loading) and pyridine (0.1 mL, 1.24 mmol) in DCM (1 mL) was added. The reaction mixture was shaken for 6 h, the solution was drained, and the resin was washed with DCM, MeOH and DCM (six times each).

A DBU solution (2% in DMF, v/v; 2 mL) was added, and the resin was shaken for 1 h. The solution was drained in a vial. An aliquot of this solution (160 μL) was diluted with acetonitrile to a total volume of 10 mL and the UV absorption of this solution was measured at 294 and 304 nm. The loading of the resin was calculated as an average of the values resulting from the formulas: $\text{Absorption}_{304\ \text{nm}} \times 16.4$/mass of resin used in mg (for 304 nm) and $\text{absorption}_{294\ \text{nm}} \times 14.2$/mass of resin used in mg (for 294 nm).

## 6. Glycosylation modules for glycosylations using the automated synthesizer

6.1. Preparation of the resin and the synthesizer for automated synthesis

The functionalized resin was loaded into the reaction vessel of the synthesizer and washed with DMF, THF, 10% acetone in DCM, and DCM (six times each with 2 mL for 25 s). The building blocks were co-evaporated with toluene three times, dissolved in DCM under an Ar atmosphere, and transferred into the vials that were placed on the corresponding port in the synthesizer. Reagents were dissolved in the corresponding solvents under an Ar atmosphere and were placed on the corresponding port in the synthesizer.

6.2. Module for glycosyl trichloroacetimidate glycosylations

The temperature of the reaction vessel was adjusted to −15 °C. For the glycosylation, a solution of glycosyl trichloroacetimidate building block **34** (3 equiv or 5 equiv in 0.75 mL DCM) was delivered to the reaction vessel followed by the addition of 0.25 mL of 50 mM TMSOTf in DCM. After 30 min the glycosylation solution was drained and the resin was washed with DCM (six times with 2 mL for 25 s). The glycosylation reaction was repeated once with 5 equiv **34** and twice with 3 equiv of **34**. At the end of the run the solid support was washed with DCM (six times with 2 mL for 25 s) and the temperature was adjusted to 25 °C.

6.3. Module for thioglycoside glycosylations

The temperature of the reaction vessel was adjusted to 25 °C. For glycosylations, a solution of thioglycoside building block **35** (3 equiv or 5 equiv in 1 mL DCM) was delivered to the reaction vessel followed by the addition of 1 mL activation solution (170 mM NIS and 17 mM TfOH in dioxane/DCM, 1:1). After 15 min, the glycosylation solution was drained and the resin was washed once with 2 mL dioxane and DCM (six times each with 2 mL for 25 s). The glycosylation reaction was repeated once for 5 equiv **35** and twice for 3 equiv of **35**. At the end of the run the solid support was washed with DCM (six times with 2 mL for 25 s) and the temperature was adjusted to 25 °C.

Other temperature conditions for thioglycoside glycosylations were explored as well. For that purpose the reaction vessel was cooled to −40 °C and the activator was added. After 5 min the temperature was adjusted to −15 °C and the reaction was performed for 40 min.

6.4. Acidic prewash before glycosylation

Prior to glycosylations using glycosyl trichloroacetimidates, the resin was washed once with 2 mL 17 mm TfOH in DCM for 2.5 min before each glycosylation reaction in each cycle.

For thioglycoside glycosylation the resin was washed one time with 2 mL activator solution (170 mM NIS and 17 mM TfOH in dioxane/DCM, 1:1) for 3.5 min before each glycosylation reaction in a cycle.

## 7. Detailed glycosylation procedures (synthesis of compounds 36–40)

7.1 Procedure to obtain compounds **36** and **37**:

Functionalized resin **25** (100 mg, loading: 0.22 mmol/g, 0.022 mmol) was loaded into the reaction vessel of the synthesizer. The temperature of the reaction vessel was adjusted to −15 °C and the resin was treated with successive washes of DMF, THF, acetone (10% in DCM) and DCM (six times, each wash with 2 mL for 25 s). For glycosylation, a solution of building block **34** (63 mg, 5 equiv, 0.11 mmol, in 0.75 mL DCM) was delivered to the reaction vessel followed by the addition of 0.25 mL of activation solution (50 mM TMSOTf in DCM). The suspension was agitated for 30 min by argon bubbling, the glycosylation solution was drained, and the resin was washed with DCM (six times, with 2 mL for 25 s). This glycosylation reaction was repeated once. In the end the temperature of the reaction vessel was adjusted to 25 °C and the resin was treated with successive washes of DCM, acetone (10% in DCM) and THF (six times, each wash with 2 mL for 25 s).

For the cleavage, the resin was removed from the reaction vessel of the synthesizer. The solid support (25 mg) was suspended in $H_2O$ (0.5 mL), and $Pd(OAc)_2$ (10 mg) and $NH_4HCO_2$ (20 mg) were added. The suspension was shaken at room temperature overnight, the solids were removed by centrifugation, and the supernatant was concentrated in vacuo. The remainder was dissolved in $H_2O$/MeCN, 1:1 and analyzed by HPLC–MS (compound **37**: ESIMS$^+$ (*m*/*z*): $[M + H]^+$ calcd for $C_{25}H_{40}N_4O_8$, 525.3; found, 525.3).

7.2 Procedure to obtain compounds **38** and **39**:

Functionalized resin **23** (50 mg, loading: 0.14 mmol/g, 7.0 µmol) was loaded into the reaction vessel of the synthesizer. The temperature of the reaction vessel was adjusted to −15 °C and the resin was treated with successive washes of DMF, THF, acetone (10% in DCM) and DCM (six times, each wash with 2 mL for 25 s). For glycosylation, a solution of building block **34** (12 mg, 3 equiv, 0.021 mmol, in 0.75 mL DCM) was delivered to the reaction vessel followed by the addition of 0.25 mL of activation solution (50 mM TMSOTf in DCM). The suspension was agitated for 30 min by argon bubbling, the glycosylation solution was drained, and the resin was washed with DCM (six times, with 2 mL for 25 s). This glycosylation reaction was repeated twice. In the end the temperature of the reaction vessel was adjusted to 25 °C and the resin was treated with successive washes of DCM, acetone (10% in DCM) and THF (six times, each wash with 2 mL for 25 s).

For the Zemplén cleavage the resin was removed from the reaction vessel of the synthesizer and was put into a syringe equipped with a filter frit. The solid support was swollen in DCM (0.9 mL), and a NaOMe solution (0.25 M in MeOH, 0.3 mL) was added. The reaction was shaken at room temperature overnight. The solution was collected and the resin was washed with DCM and DCM/MeOH, 1:1 (six times, each wash with 2 mL). The solutions were combined and neutralized with IR-120 ($H^+$) and the solvents were removed in vacuo. The remainder was dissolved in a $H_2O$/MeCN, 1:1 and analyzed by HPLC–MS (compound **39**: ESIMS$^+$ (*m/z*): $[M + H]^+$ calcd for $C_{54}H_{62}N_6O_{11}$, 971.5; found, 971.1).

7.3 Procedure to obtain compounds **40**:

Functionalized resin **23** (50 mg, loading: 0.14 mmol/g, 7.0 µmol) was loaded into the reaction vessel of the synthesizer. The temperature of the reaction vessel was adjusted to −15 °C and the resin was treated with successive washes of DMF, THF, acetone (10% in DCM) and DCM (six times, each wash with 2 mL for 25 s). For glycosylation, a solution of building block **34** (12 mg, 3 equiv, 0.021 mmol, in 0.75 mL DCM) was delivered to the reaction vessel followed by the addition of 0.25 mL of activation solution (50 mM TMSOTf in DCM). The suspension was agitated for 30 min by argon bubbling, the glycosylation solution was drained, and the resin was washed with DCM (six times, with 2 mL for 25 s). This glycosylation reaction was repeated twice. In the end the temperature of the reaction vessel was adjusted to 25 °C, and the resin was washed with DCM and THF (six times, each wash with 2 mL for 25 s).

In the next step, Staudinger solution (2 mL; 0.5 M $PMe_3$ in $THF/H_2O/NEt_3$: 18.75/1/0.25) was added to the resin, which was incubated for 30 min at 25 °C. After the reaction the solution was drained, and the resin was washed with THF (six times, with 2 mL for 25 s). The reaction was repeated twice.

For the Zemplén cleavage the resin was removed from the reaction vessel of the synthesizer and was put into a syringe equipped with a filter frit. The solid support was swollen in DCM (1.8 mL), and a NaOMe solution (0.25 M in MeOH, 0.5 mL) was added. The reaction was shaken at room temperature overnight. The solution was collected and the resin was washed with DCM and DCM/MeOH, 1:1 (six times, each wash with 2 mL). The solutions were combined and neutralized with IR-120 ($H^+$) and the solvents were removed in vacuo. The remainder was dissolved in $H_2O$/MeCN, 1:1 and analyzed by HPLC–MS (compound **40**: ESIMS$^+$ (*m/z*): $[M + H]^+$ calcd for $C_{54}H_{64}N_4O_{11}$, 945.5; found, 944.5).

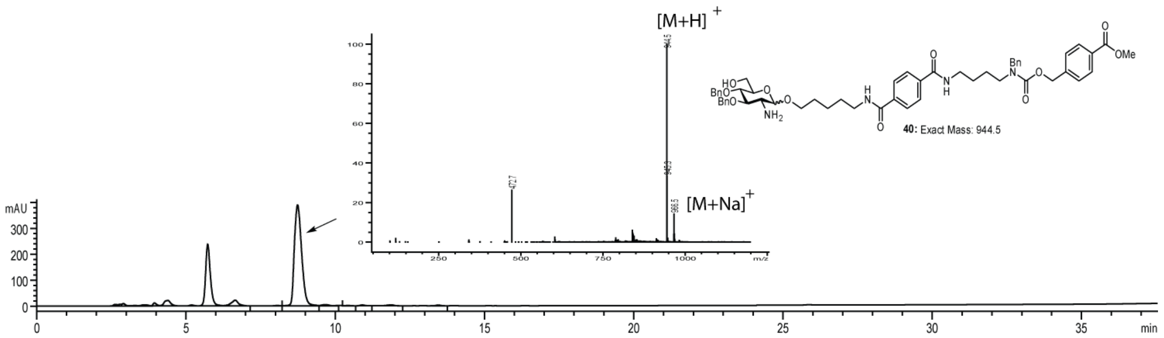


**Figure S3:** LC–MS chromatogram after automated Staudinger reduction. (Nucleosil C4, 40% → 80% in 30 min, eluents: $H_2O$ and MeCN, detection at 254 nm).

## 8. Spectra of new compounds

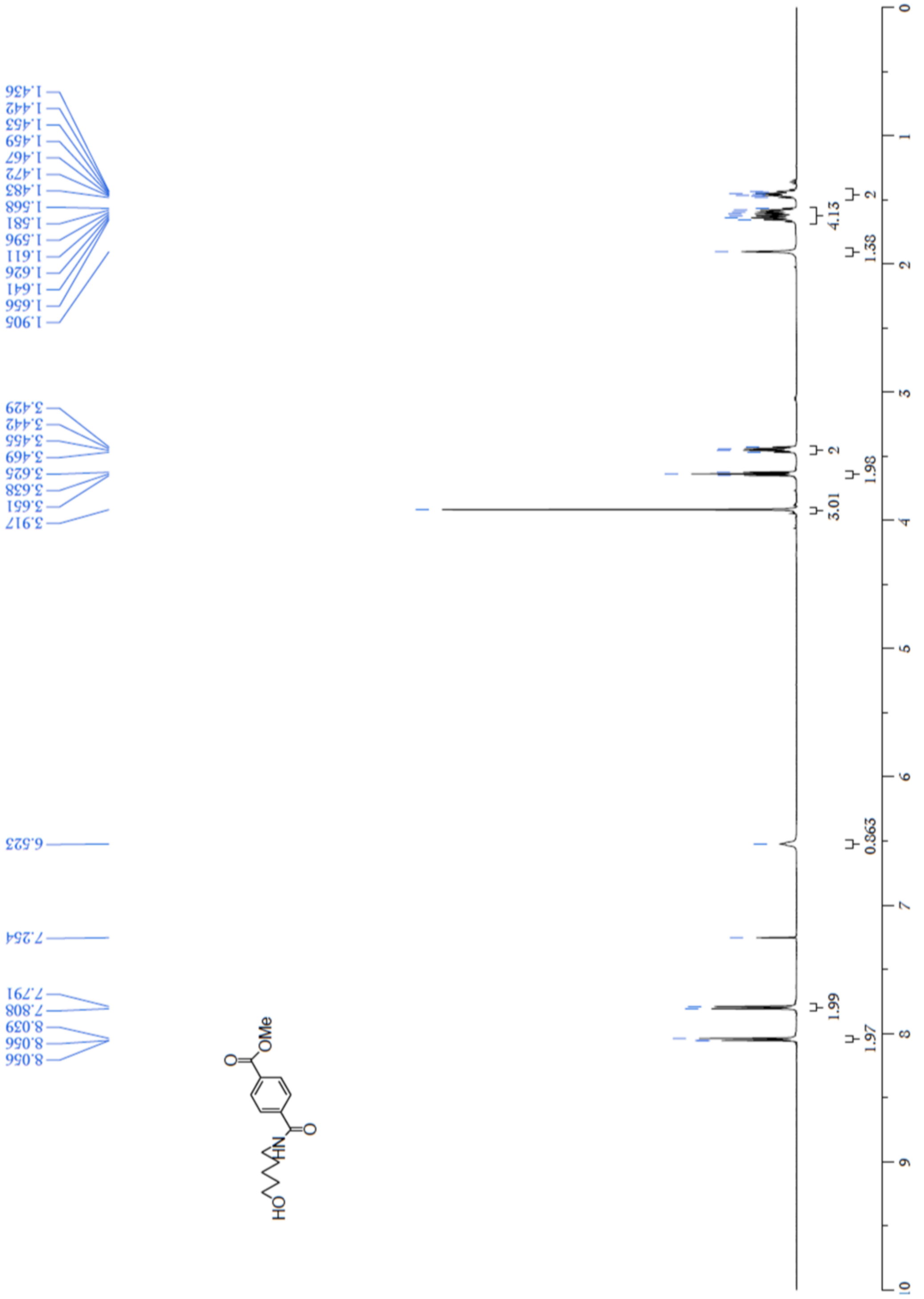

**Figure S4:** $^1$H NMR of compound **5**.

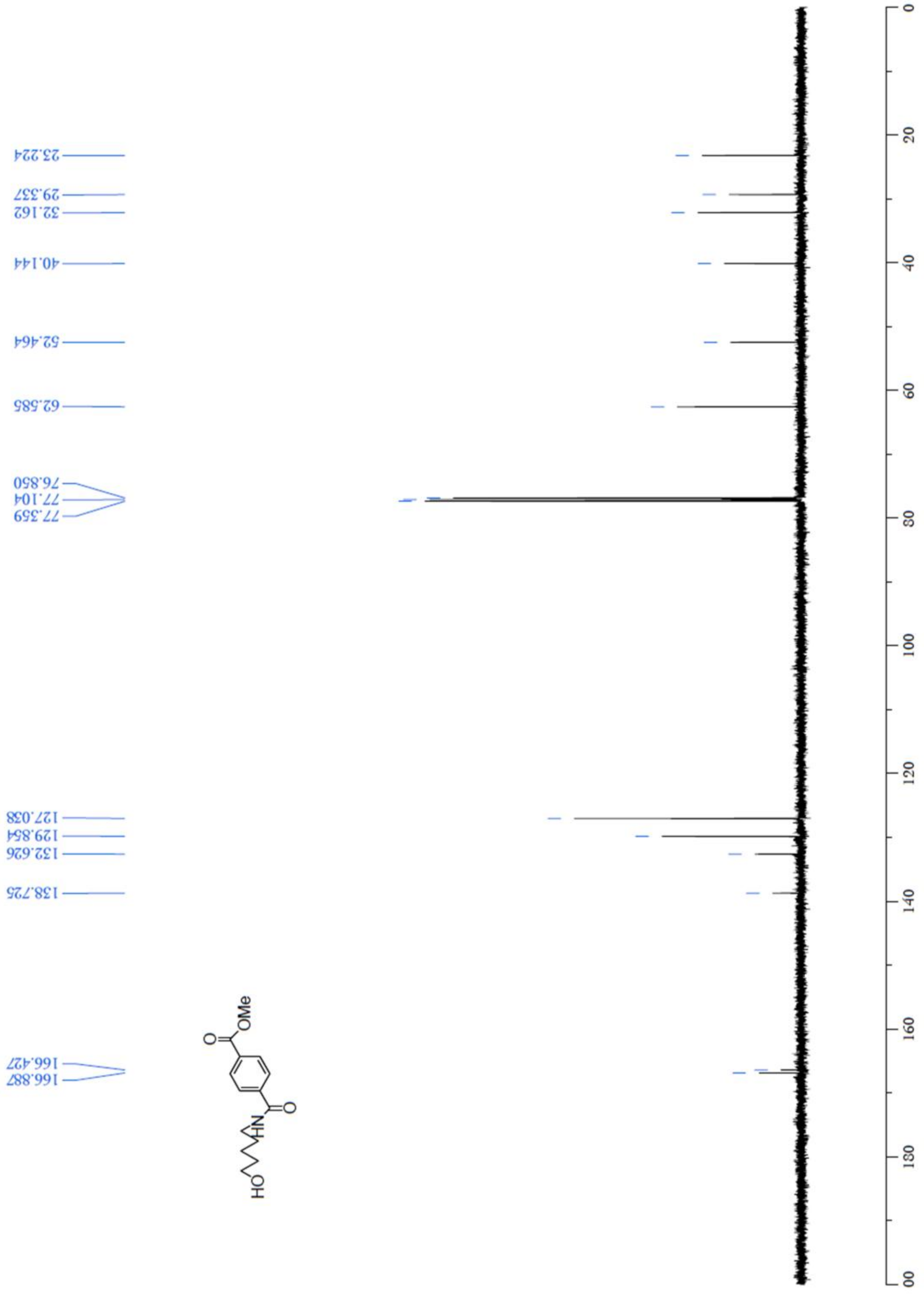


**Figure S5:** $^{13}$C NMR of compound **5**.

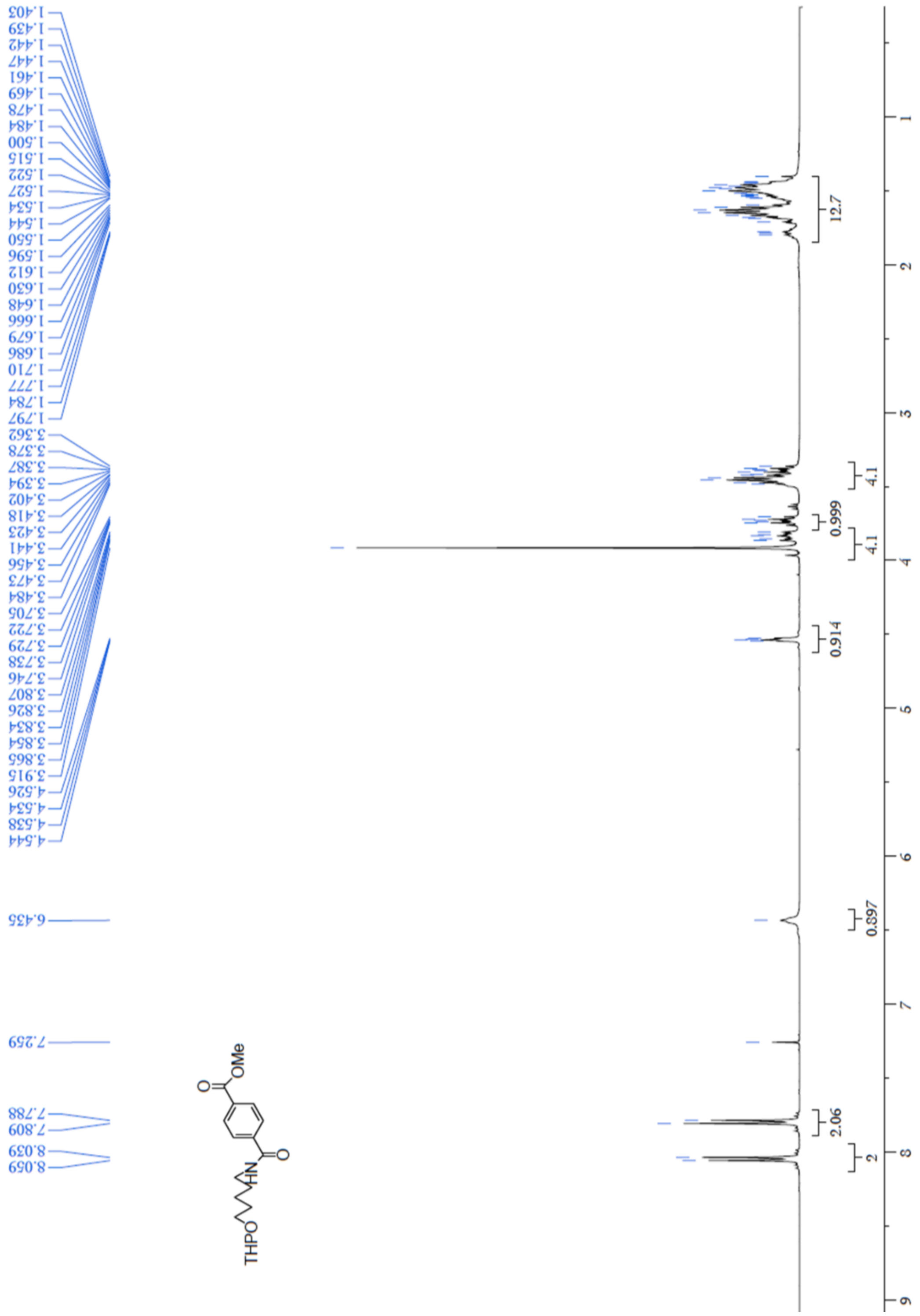


**Figure S6:** ¹H NMR of compound **6**.

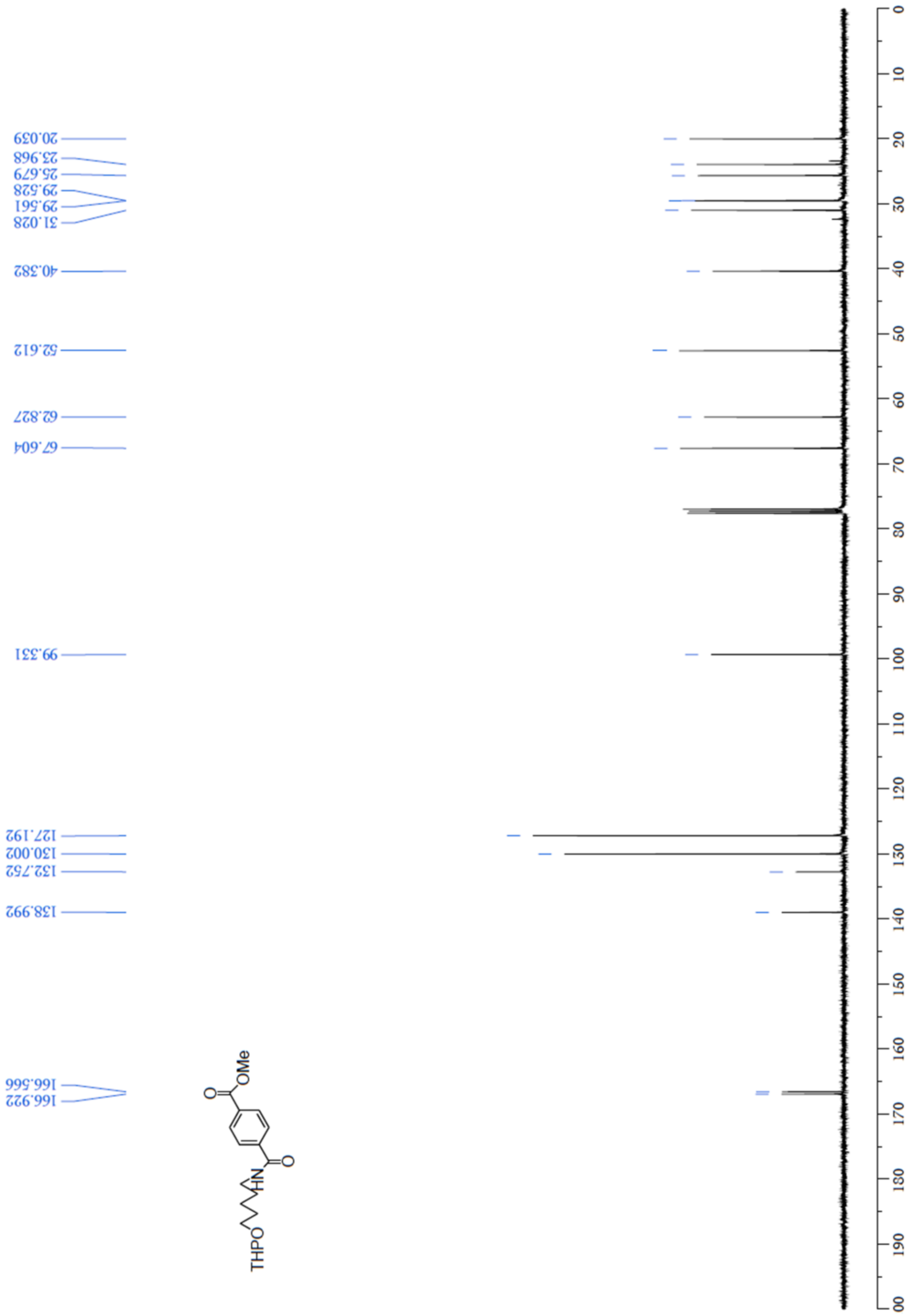

**Figure S7:** $^{13}$C NMR of compound **6**.

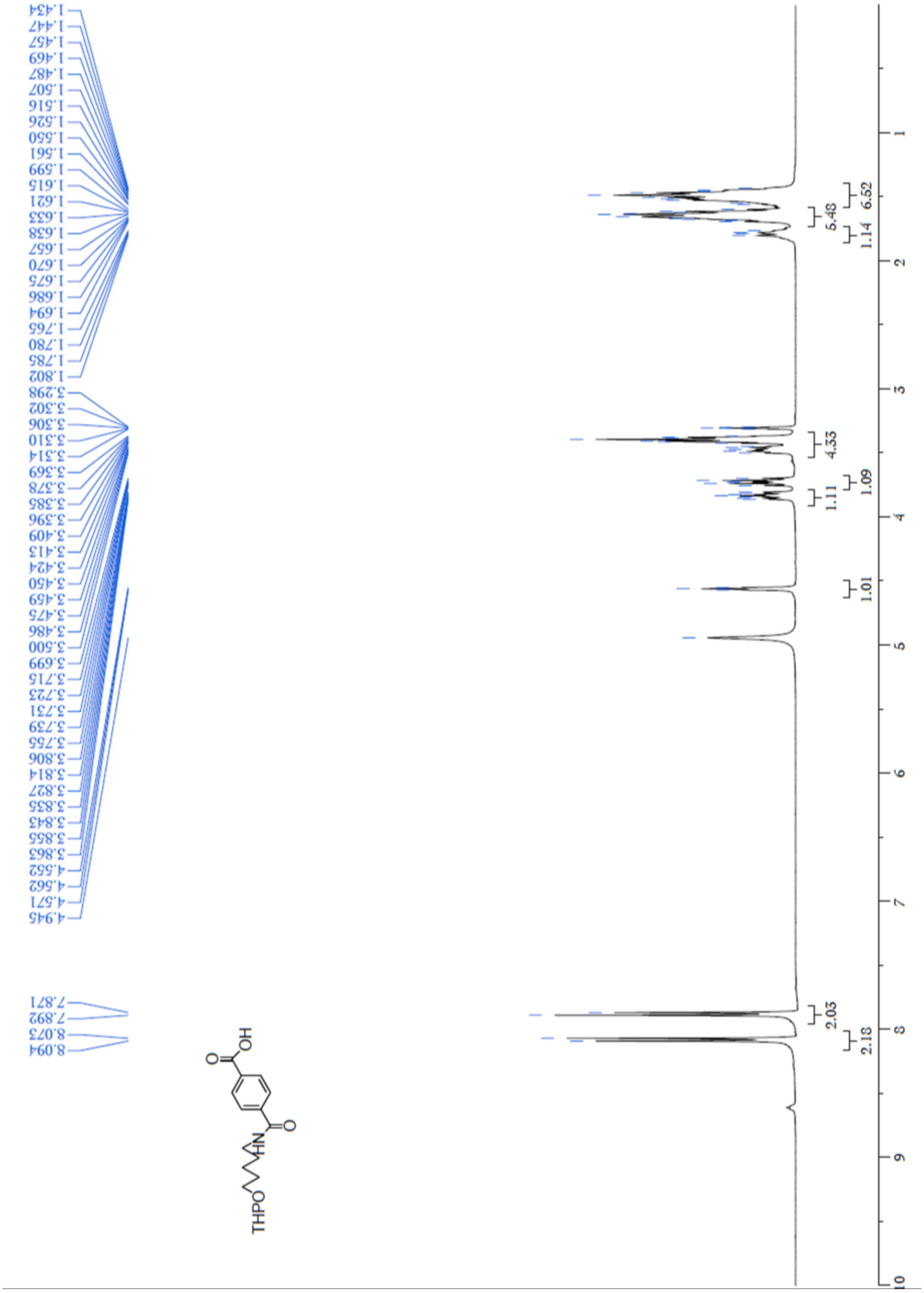


**Figure S8:** $^{1}$H NMR of compound **7**.

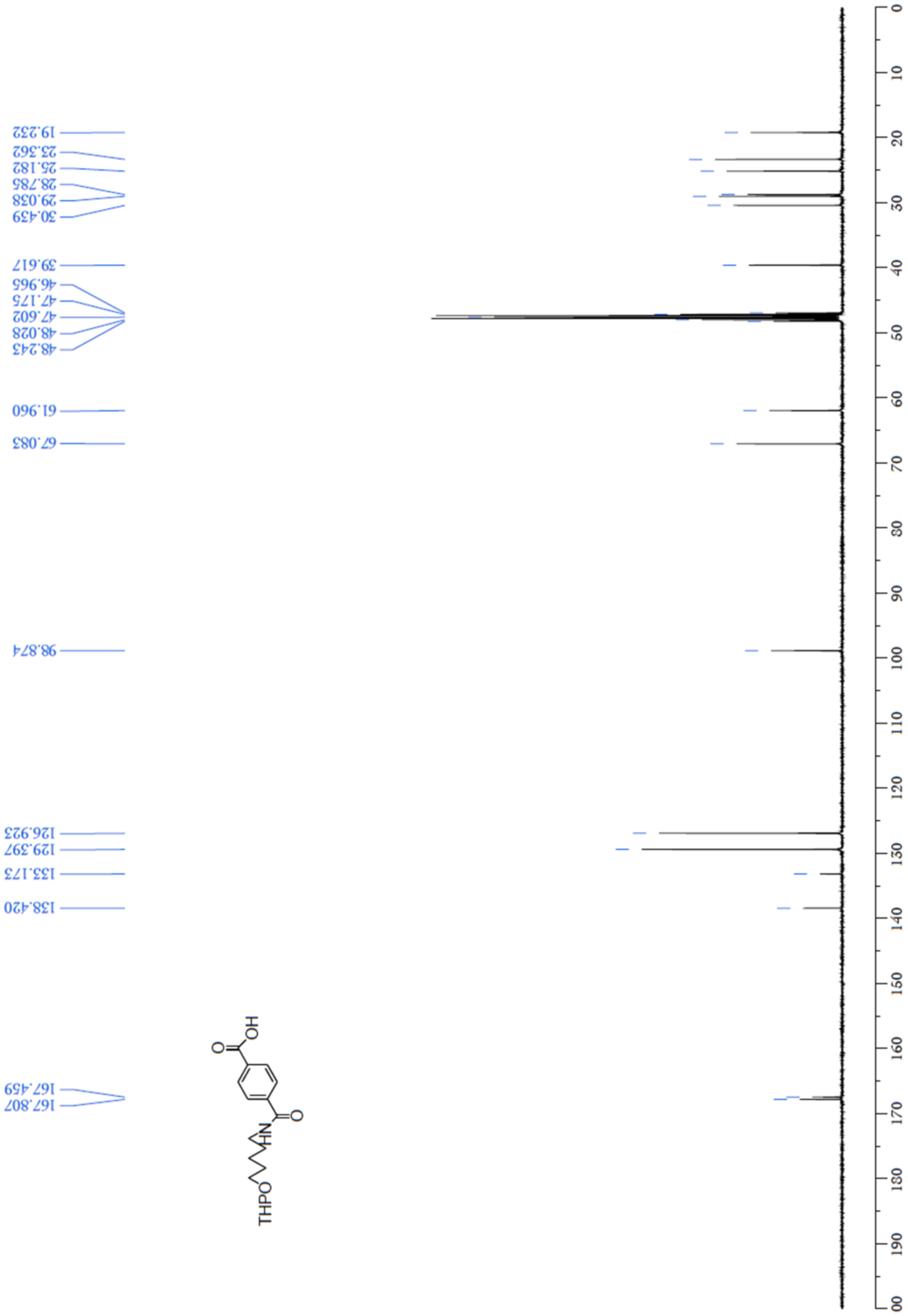


**Figure S9:** $^{13}$C NMR of compound **7**.

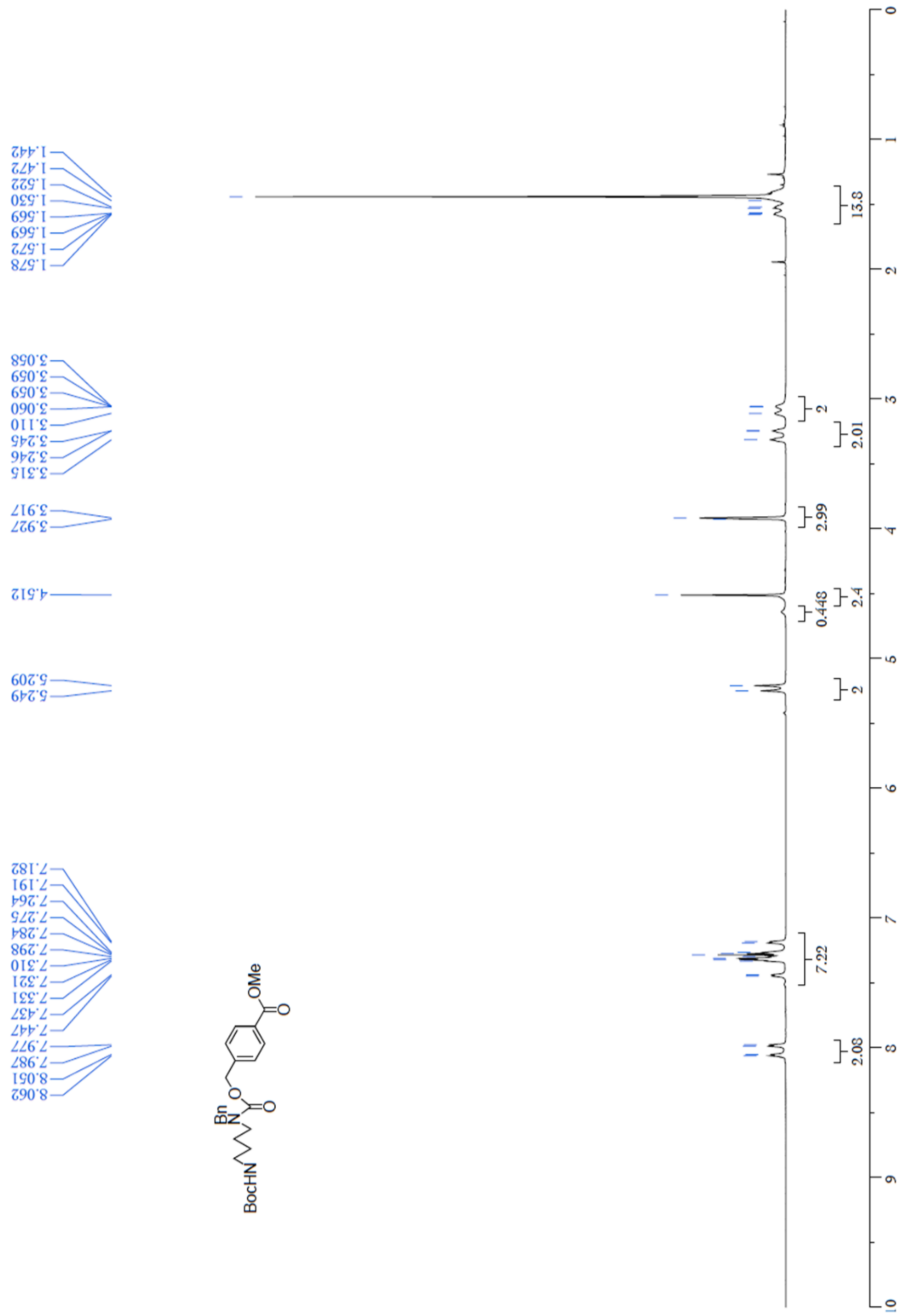


**Figure S10:** $^{1}$H NMR of compound **11**.

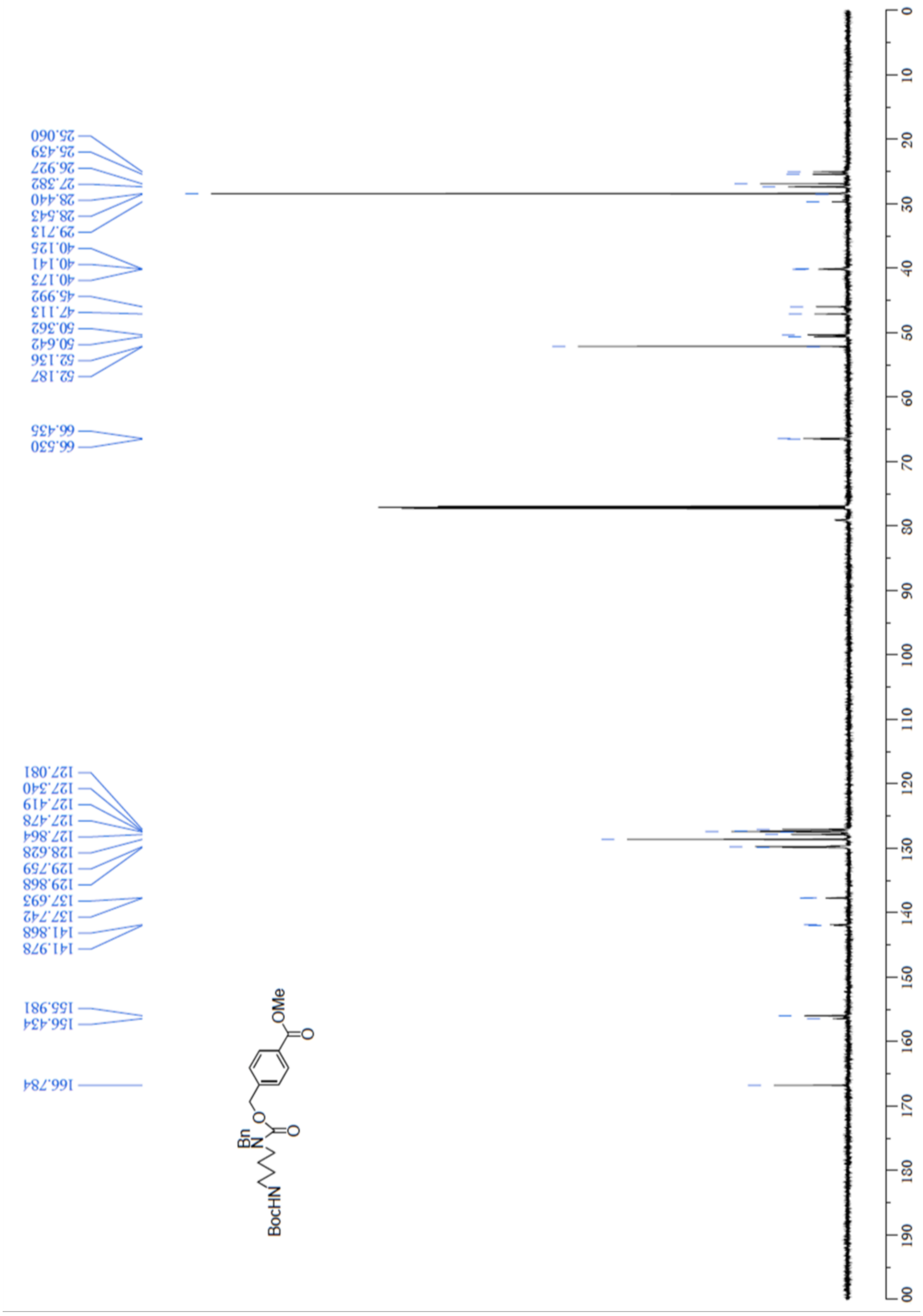


**Figure S11:** $^{13}$C NMR of compound **11**.

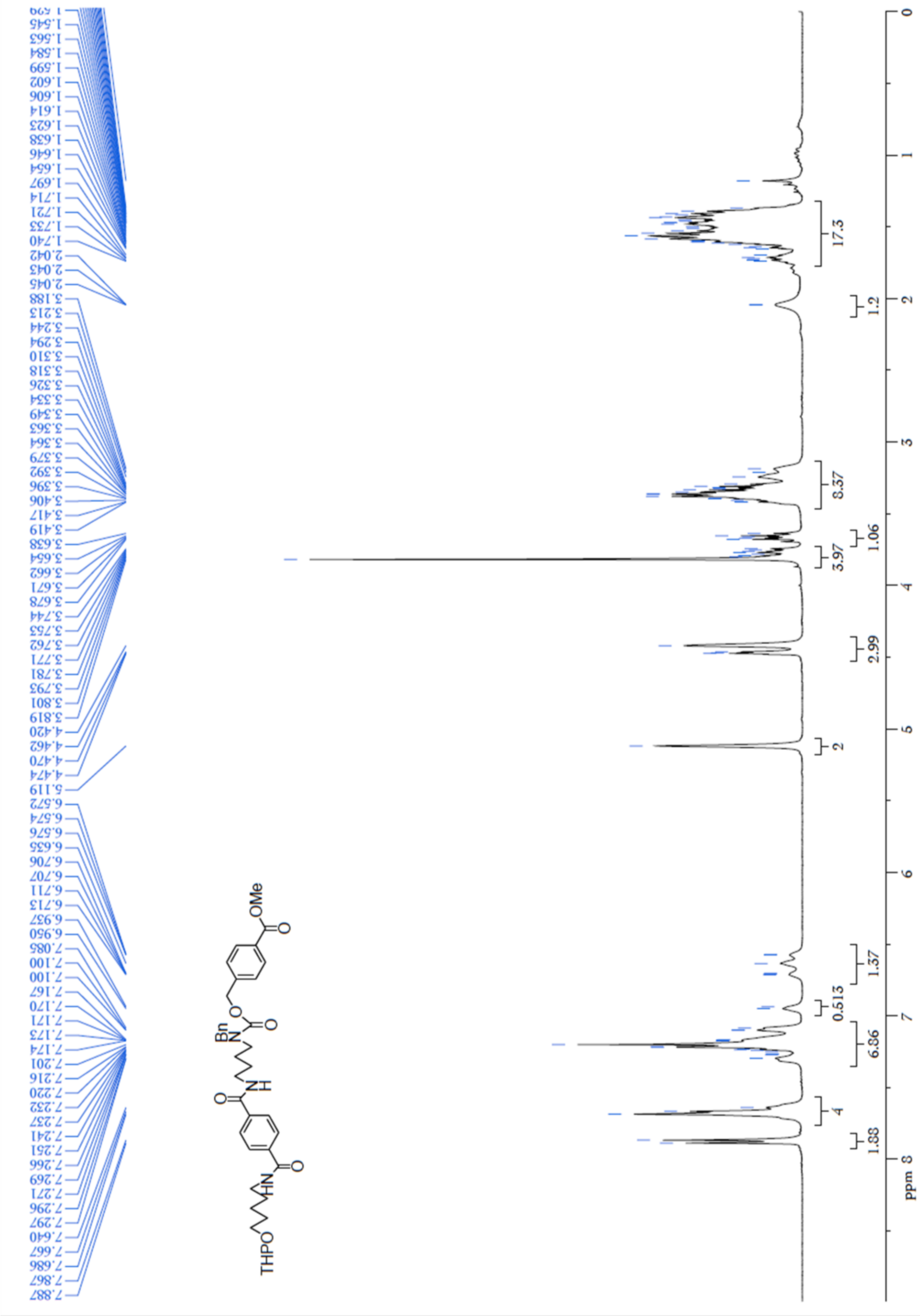

**Figure S12:** $^{1}$H NMR of compound **13**.

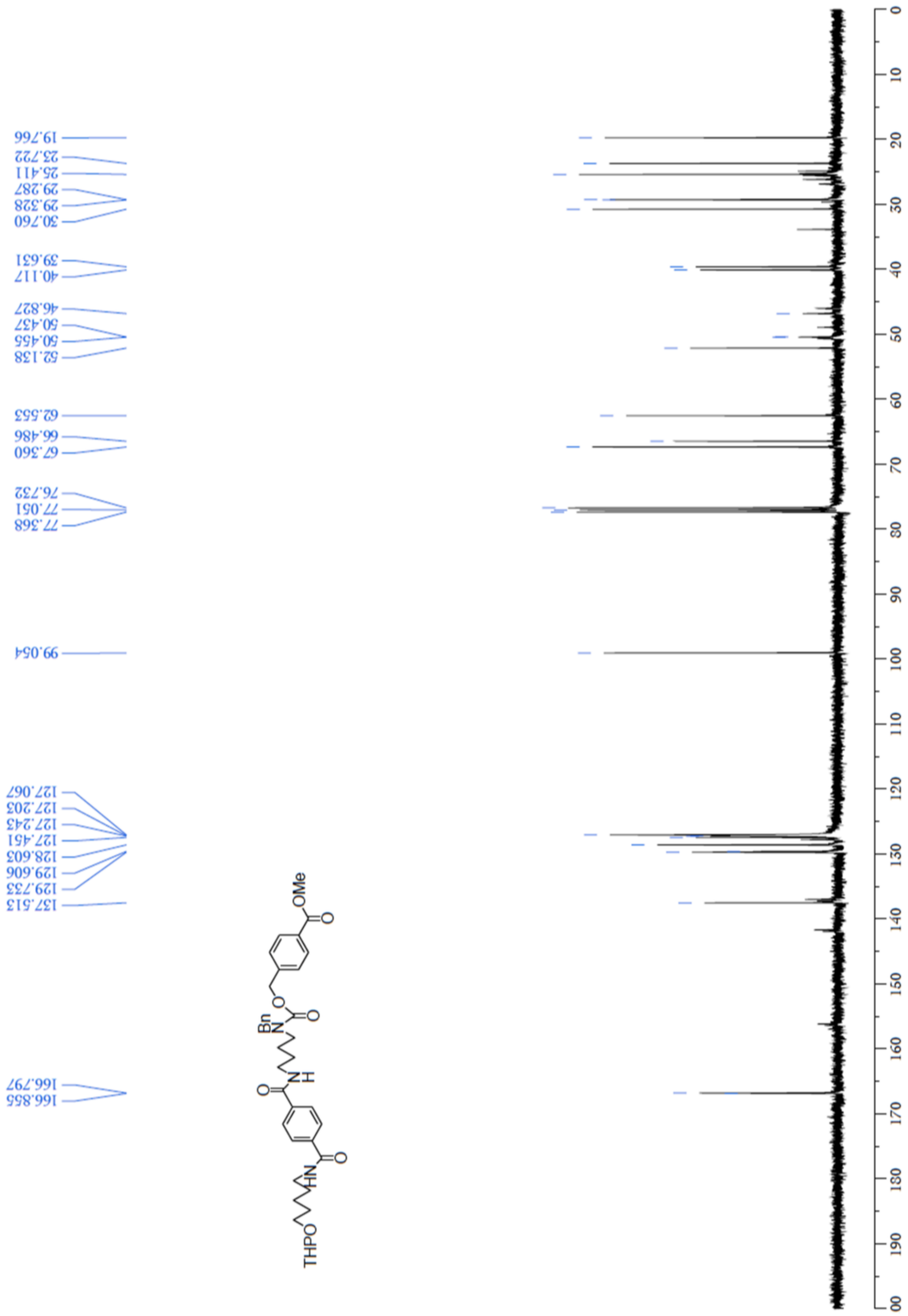


**Figure S13:** $^{13}$C NMR of compound **13**.

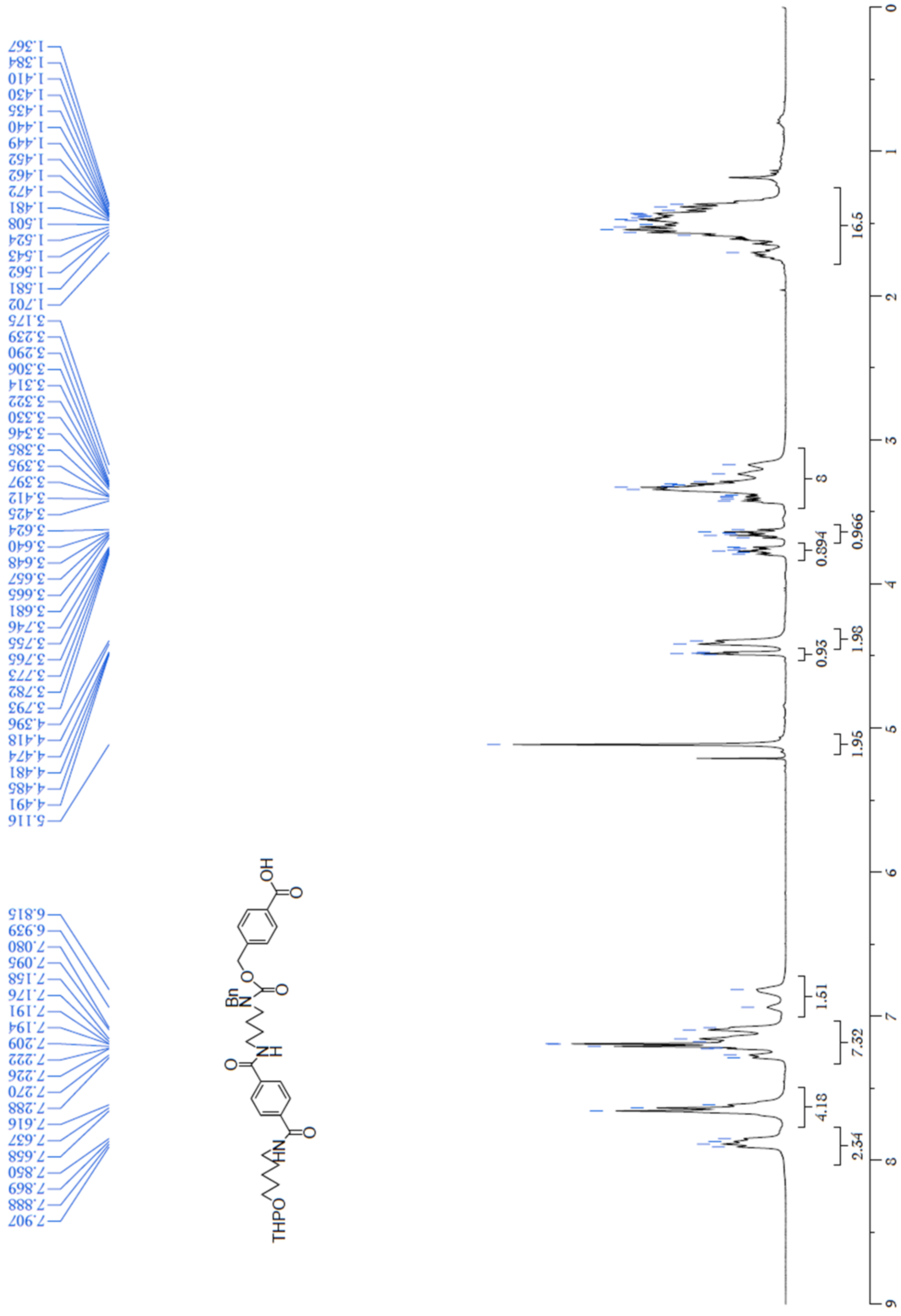


**Figure S14:** $^{1}$H NMR of compound **1**.

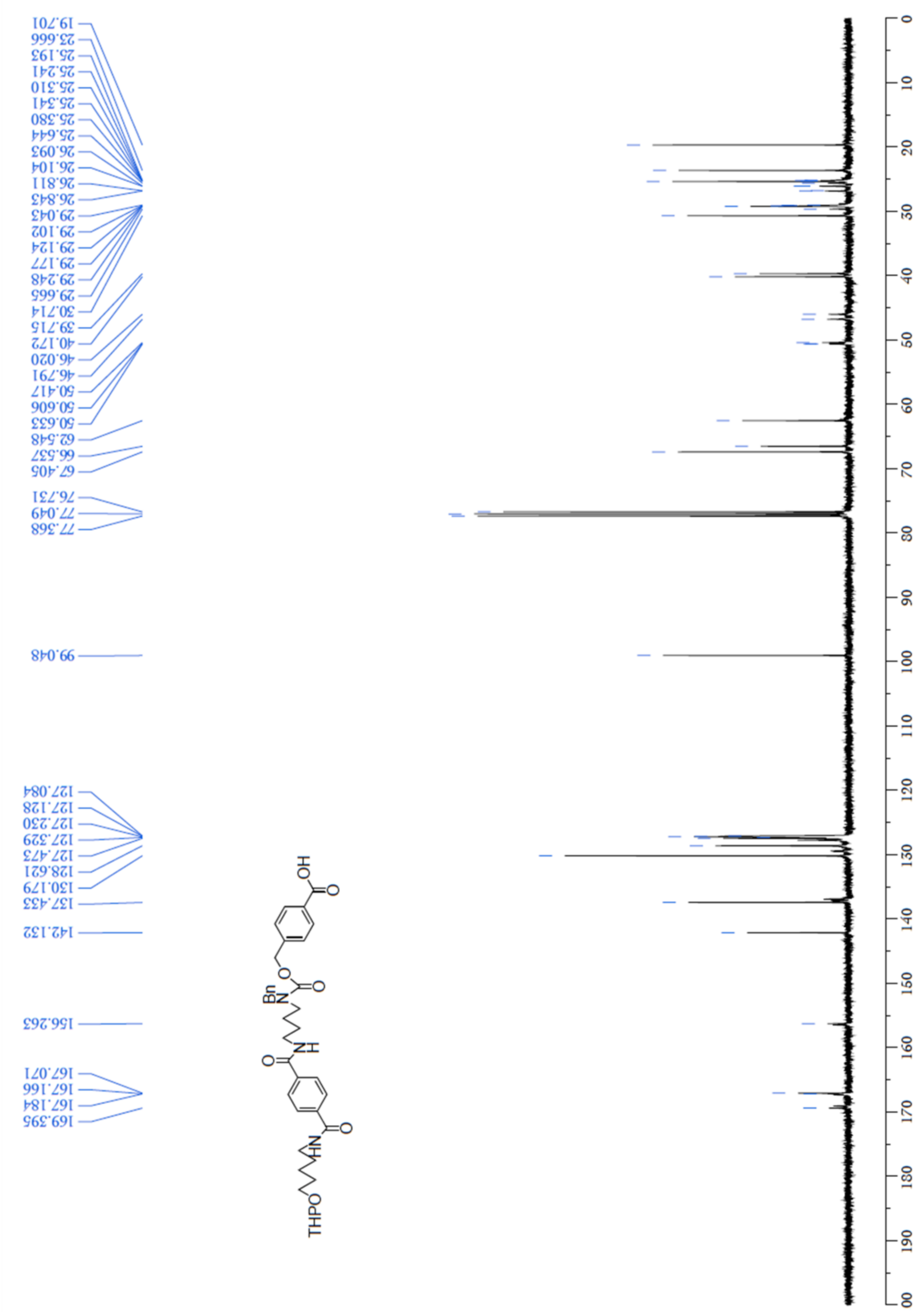


**Figure S15:** $^{13}$C NMR of compound **1**.

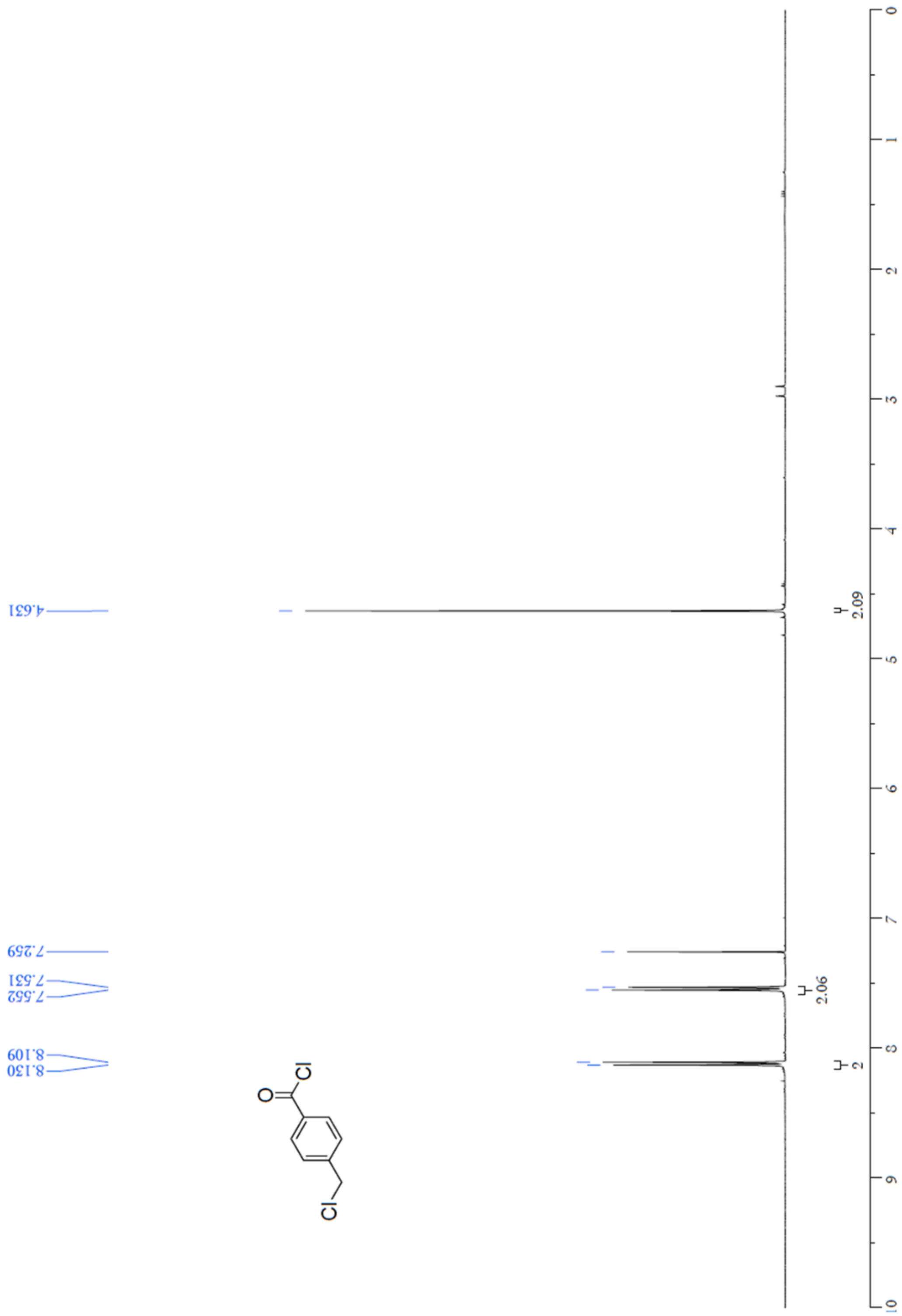


**Figure S16:** $^{1}$H NMR of compound **2**.

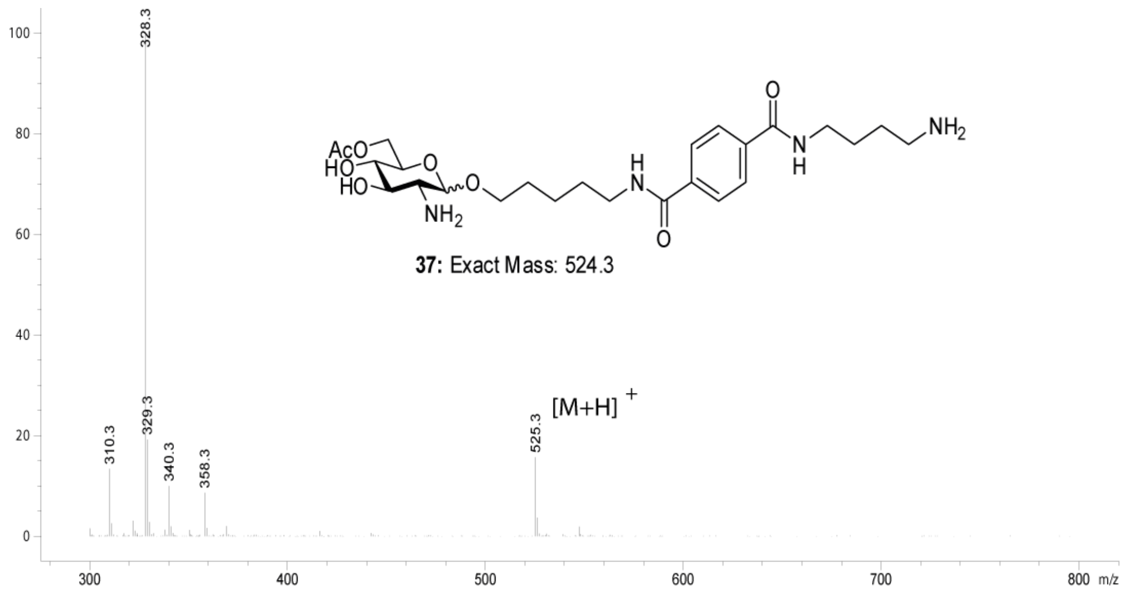


**Figure S17:** MS spectrum of compound **37** (taken from LCMS).

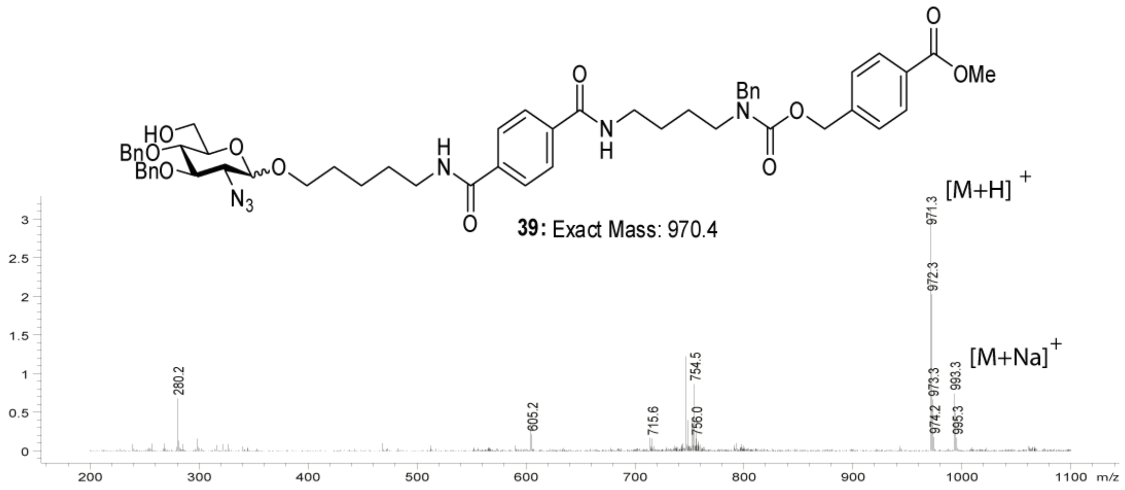


**Figure S18:** MS spectrum of compound **39** (taken from LCMS).
